# Interfacial Coordination‐Engineered Emulgel Modulates the Osteo‐Immune Microenvironment in Periodontitis Treatment Via HIF1 Signaling Pathway‐Induced Metabolic Reprogramming

**DOI:** 10.1002/advs.202509998

**Published:** 2025-09-29

**Authors:** Hui Zhang, Xiao Huang, Rui Chen, Weimin Lin, Linke Li, Xi Peng, Xinxi Yang, Xingyu Chen, Tailin Guo, Jie Weng, Jianshu Li, Quan Yuan, Huan Tan, Mengyuan Wang

**Affiliations:** ^1^ College of Medicine Southwest Jiaotong University Chengdu Sichuan 610031 P. R. China; ^2^ Center of Obesity and Metabolic Diseases Department of General Surgery The Third People's Hospital of Chengdu Chengdu 610014 P. R. China; ^3^ Key Laboratory of Advanced Technologies of Materials Ministry of Education School of Materials Science and Engineering Southwest Jiaotong University Chengdu 610031 P. R. China; ^4^ Department of stomatology The Third People's Hospital of Chengdu The Affiliated Hospital of Southwest Jiaotong University Chengdu Sichuan 610031 P. R. China; ^5^ State Key Laboratory of Oral Diseases and National Center for Stomatology National Clinical Research Center for Oral Diseases West China Hospital of Stomatology Chengdu 610041 P. R. China; ^6^ College of Polymer Science and Engineering. State Key Laboratory of Polymer Materials Engineering Sichuan University Chengdu 610041 P. R. China

**Keywords:** HIF‐1 signaling pathway, high internal phase pickering emulsion, macrophage polarization, metabolic reprogramming, periodontitis, quercetin

## Abstract

A multifunctional high internal phase Pickering emulsion (HIPPE‐QU) is developed to address immune dysregulation in periodontitis by reprogramming macrophage metabolism and promoting tissue regeneration. Fabricated via one‐step homogenization, HIPPE‐QU features a 75% internal phase and is co‐stabilized by gelatin nanoparticles (GNPs) and copper‐doped hydroxyapatite (HA‐Cu), forming a gel‐like, injectable emulgel suitable for clinical application. Hydrophobic quercetin (QU) is encapsulated in the internal phase and chelates with Cu^2^⁺ and Ca^2^⁺ at the interface, enabling sustained and pH‐responsive release under the acidic periodontal environment. The spatially organized loading strategy across dispersed, interfacial, and internal phases yields synergistic antibacterial and immunoregulatory effects. Mechanistically, HIPPE‐QU modulates macrophage glycolysis and ATP metabolism via HIF‐1 signaling, suppresses ROS overproduction, and promotes M1‐to‐M2 polarization. In vivo, HIPPE‐QU alleviates inflammation, restores BMSC osteogenic capacity, and enhances periodontal tissue regeneration. This work presents an innovative immune‐reprogramming platform with translational potential for precision modulation of the periodontal immune microenvironment.

## Graphical abstract

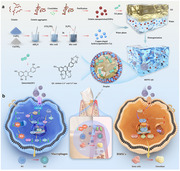

## 1 Introduction

Periodontitis is a chronic inflammatory disease initiated by microbial dysbiosis and exacerbated by the host immune response. This leads to destruction of periodontal tissues, making it a leading cause of tooth loss in adults.^[^ ^1^ ^]^ Periodontitis affects nearly half the global population, including over one billion severe cases,^[^ ^2^ ^]^ and is linked to increased risk of systemic diseases, including cardiovascular disorders, diabetes, and Alzheimer's.^[^ ^3^ ^]^ However, mechanical debridement and antibiotic therapy eliminate pathogenic bacteria but fail to mitigate host‐driven tissue destruction, underscoring the need to modulate host immunity.^[^ ^4^ ^]^ Immunomodulatory strategies are emerging, including multifunctional biomaterials conferring antibacterial and immunomodulatory effects while promoting periodontal tissue regeneration. Macrophages centrally regulate the periodontal immune microenvironment, orchestrating pathogen defense, immunomodulation, and host‐microbiota homeostasis.^[^ ^5^ ^]^ Upon microbial challenge (e.g., LPS) stimulation, they polarize to the pro‐inflammatory M1 phenotype, undergoing metabolic reprogramming characterized by enhanced aerobic glycolysis and altered mitochondrial metabolism.^[^ ^3a^ ^]^ This shift upregulates ATP‐citrate lyase (Acly) and hypoxia‐inducible factor‐1α (HIF‐1α), driving augmented phagocytosis and excessive reactive oxygen species (ROS) production.^[^ ^6^ ^]^ Furthermore, HIF‐1 and Toll‐like receptor cascades induce pro‐inflammatory mediators (IL‐1β, TNF‐α, iNOS), perpetuating an inflammatory loop that exacerbating tissue damage.^[^ ^7^ ^]^ Consequently, targeting macrophage metabolism—suppressing glycolysis and ROS while promoting M1‐to‐M2 repolarization—emerges as a strategy to break this inflammatory cycle and foster tissue repair.

Quercetin (QU), a liposoluble a lipophilic natural phenolic flavonoid with hydroxyl groups, a 2,3‐double bond, and catechol moieties, exhibits strong ROS‐scavenging and anti‐inflammatory properties.^[^ ^8^ ^]^ QU has been widely studied for chronic inflammatory conditions, where it modulates macrophage metabolic reprogramming by regulating glycolysis.^[^ ^9^ ^]^ Xu et al. further reported that QU enhances periodontal bone regeneration by mitigating oxidative stress.^[^ ^10^ ^]^ However, its precise immunomodulatory role in periodontitis—including effects on macrophage metabolism, ROS production, and interactions with bone marrow–derived mesenchymal stem cells (BMSCs)—remains unclear. Moreover, the poor aqueous solubility, limited bioavailability, and chemical instability of QU significantly restrict its clinical utility, thereby highlighting the need for localized delivery systems.^[^ ^11^ ^]^ The irregular anatomical shape of periodontal pockets and the chronicity of periodontitis demand an injectable, biocompatible carrier for local drug delivery.^[^ ^12^ ^]^ Hydrogels have been explored for this purpose, but their complex polymer preparation and limited capacity for hydrophobic drugs (such as QU) constrain clinical application.^[^ ^13^ ^]^ In contrast, Pickering emulsions, a novel class of multiphase systems stabilized by nano‐ or micro‐sized solid particles instead of conventional surfactants, offer an attractive alternative.^[^ ^14^ ^]^ Because particles adsorb irreversibly at the oil–water interface, these emulsions require far lower stabilizer concentrations and thus reduce cytotoxicity.^[^ ^15^ ^]^ Solid particles could develop strong interactions at interfaces to form rigid layers, which endow droplets with outstanding stability toward coalescence, Ostwald ripening, and creaming.^[^ ^16^ ^]^ High internal phase Pickering emulsion (HIPPE, internal phase fraction > 74%), also defined as emulgel, features closely packed droplets that confer gel‐like consistency and high viscosity while maintaining excellent fluidity.^[^ ^17^ ^]^ These properties make emulgel an ideal injectable depot for periodontal pockets, which promote tight filling in irregular periodontal defects. Moreover, this platform simultaneously carries multiple therapeutic agents in compartmentalized aqueous and oil phases, enabling synergistic treatment with hydrophilic and hydrophobic bioactive compounds, such as QU.

In order to repair the complex periodontal microenvironment, the antibacterial treatment and the regeneration of the alveolar bone are also key issues to be considered. Hydroxyapatite (HA), the primary inorganic substance of human bones and teeth, confers essential biocompatibility, osteoconductivity, and biological activity to the mature bone matrix. To expand the functionality of HA, many studies have confirmed that trace elements‐doped HA can control the degradation of calcium phosphate materials and endow them with additional biological functions.^[^ ^18^ ^]^ Among these, Cu‐doped HA (HA‐Cu) displays excellent comprehensive properties involving broad‐spectrum antibacterial, osteogenic activities, and angiogenic effects.^[^ ^19^ ^]^ However, free nanoparticles are easily endocytosed induced by clathrin‐meditated endocytosis, and possibly generate ROS in lysosomes, which causes cell death. Meanwhile, once external Cu introduction disrupts the internal Cu homeostasis, Cu^2^⁺ is capable of inducing oxidative stress, apoptosis through Fenton‐like reactions, and is able to trigger cuproptosis by causing the aggregation of mitochondrial proteins.^[^ ^20^ ^]^ To harness Cu^2^⁺ safely, the immobilization of HA‐Cu and the controlled release of Cu^2^⁺ is important for maintaining sustained therapeutic levels in the gingival sulcus.^[^ ^21^ ^]^ The anchor of nanoparticle on the interface of the emulsion droplet always has a large free energy of desorption,^[^ ^22^ ^]^ which therefore forms a strong mechanical barrier around the interface, thereby immobilizing the particles as well as preventing the droplets from merging. However, HA‐Cu particles alone have poor interfacial wettability to migrate to the interface, hindering HIPPE stabilization. Our previous finding demonstrated that spherical gelatin nanoparticles (GNPs) exhibit pronounced deformation capacity and surface wettability, which could effectively stabilize HIPPE.^[^ ^23^ ^]^ With the aid of GNPs, HA‐Cu can behave as co‐stabilizers to migrate to the O/W interface, and then be trapped and immobilized, which could achieve controlled release of copper ions. This combined stabilizer strategy yielded a novel therapeutic HIPPE integrating antibacterial, bone‐regenerative, and immunomodulatory effects for periodontitis treatment.

**Scheme 1:**
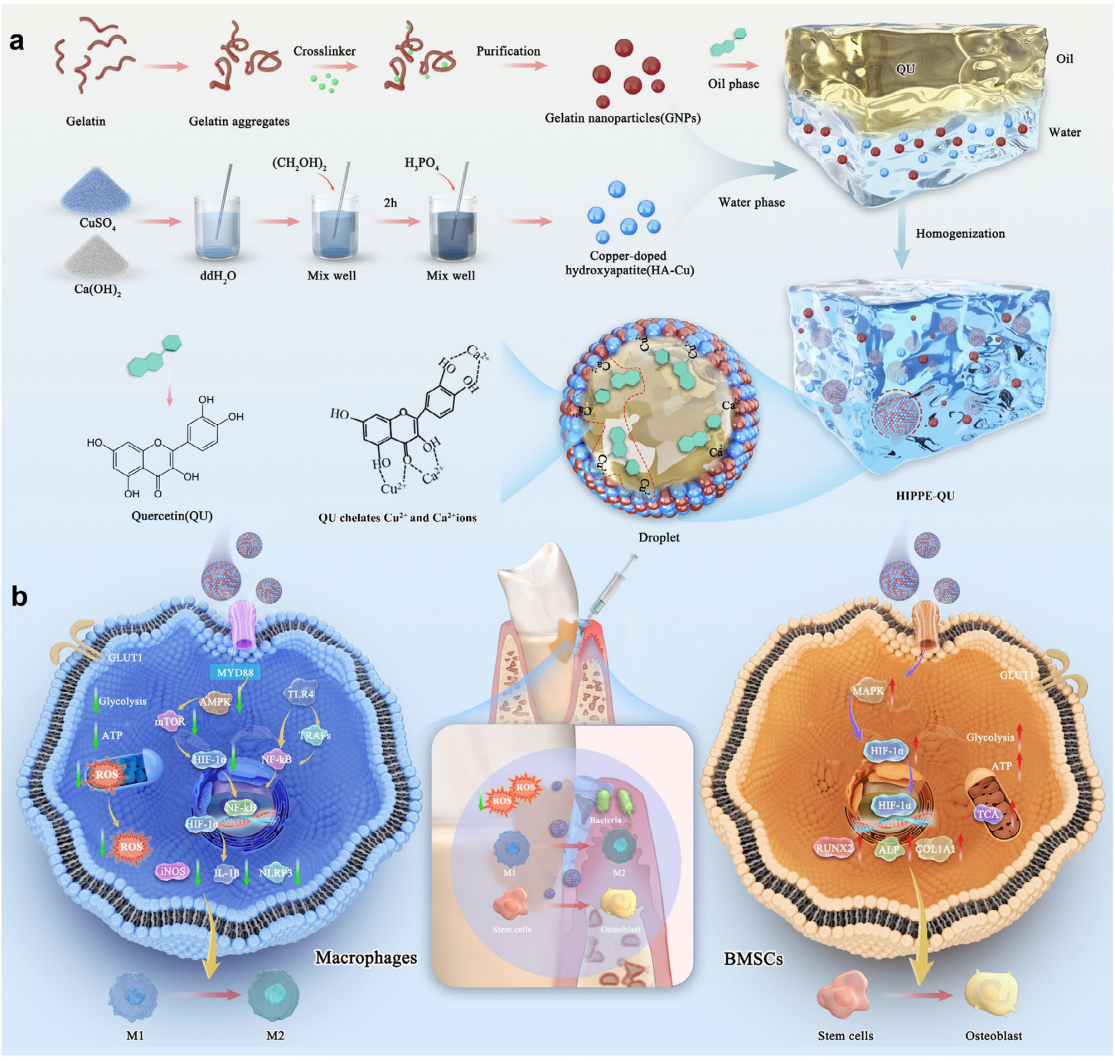
Schematic illustration of HIPPE‐QU‐mediated periodontal regeneration. a) Fabrication process and microstructure of HIPPE‐QU. b) Antimicrobial and immunomodulatory HIPPE‐QU hydrogel for periodontal regeneration. HIPPE‐QU hydrogel co‐delivers HA‐Cu, GNPs, and QU, providing antimicrobial effects and reprogramming the immune microenvironment to promote periodontal healing. It modulates macrophage glycolysis and reduces ROS via HIF‐1 signaling, drives M1‐to‐M2 polarization, and fosters a bone‐regenerative microenvironment for enhanced periodontal repair.

Herein, we formulated an oil‐in‐water HIPPE co‐stabilized by GNPs and HA‐Cu using one‐step homogenization without any complicated chemical modification. We loaded the hydrophobic polyphenol QU into the oil phase to create a QU‐loaded emulgel (denoted as HIPPE‐QU), leveraging its immune reprogramming properties. Natural flavonoids possess functional groups such as the ketone and phenolic hydroxyl, allowing them to coordinate with metal ions.^[^ ^24^ ^]^ At the oil–water interface, ketone and phenolic hydroxyl in QU could chelate with Cu^2^⁺ and Ca^2^⁺ from HA‐Cu, forming interfacial coordination complexes that govern release kinetics. This chelation ensured controlled, sustained co‐release of QU and Cu^2^⁺, maintaining therapeutic levels at the periodontal site. Moreover, the acidic periodontal microenvironment triggered partial emulsion coalescence, accelerating QU release. HIPPE‐QU behaved as a gel‐like viscoelastic fluid that was readily injectable and exhibited prolonged sustained‐release behavior, meeting key handling requirements for direct clinical application into periodontal pockets. By co‐loading active components in the dispersed phase (HA‐Cu), interface (GNPs, HA‐Cu, and chelation complexes), and internal phase (QU), this system achieved synergistic antimicrobial activity and immune reprogramming to promote osteogenesis and periodontal healing. Mechanistically, HIPPE‐QU reprogrammed macrophages by attenuating pro‐inflammatory metabolic activity—reducing glycolysis and ROS generation, restoring ATP metabolism through the HIF‐1 signaling pathway—and promoting M1‐to‐M2 polarization, thereby creating a regenerative microenvironment conducive to bone regeneration and periodontal repair (**Scheme** **1**).

## 2 Results

### 2.1 Mechanism of Macrophage Polarization and Metabolism in Periodontitis

**Figure 1:**
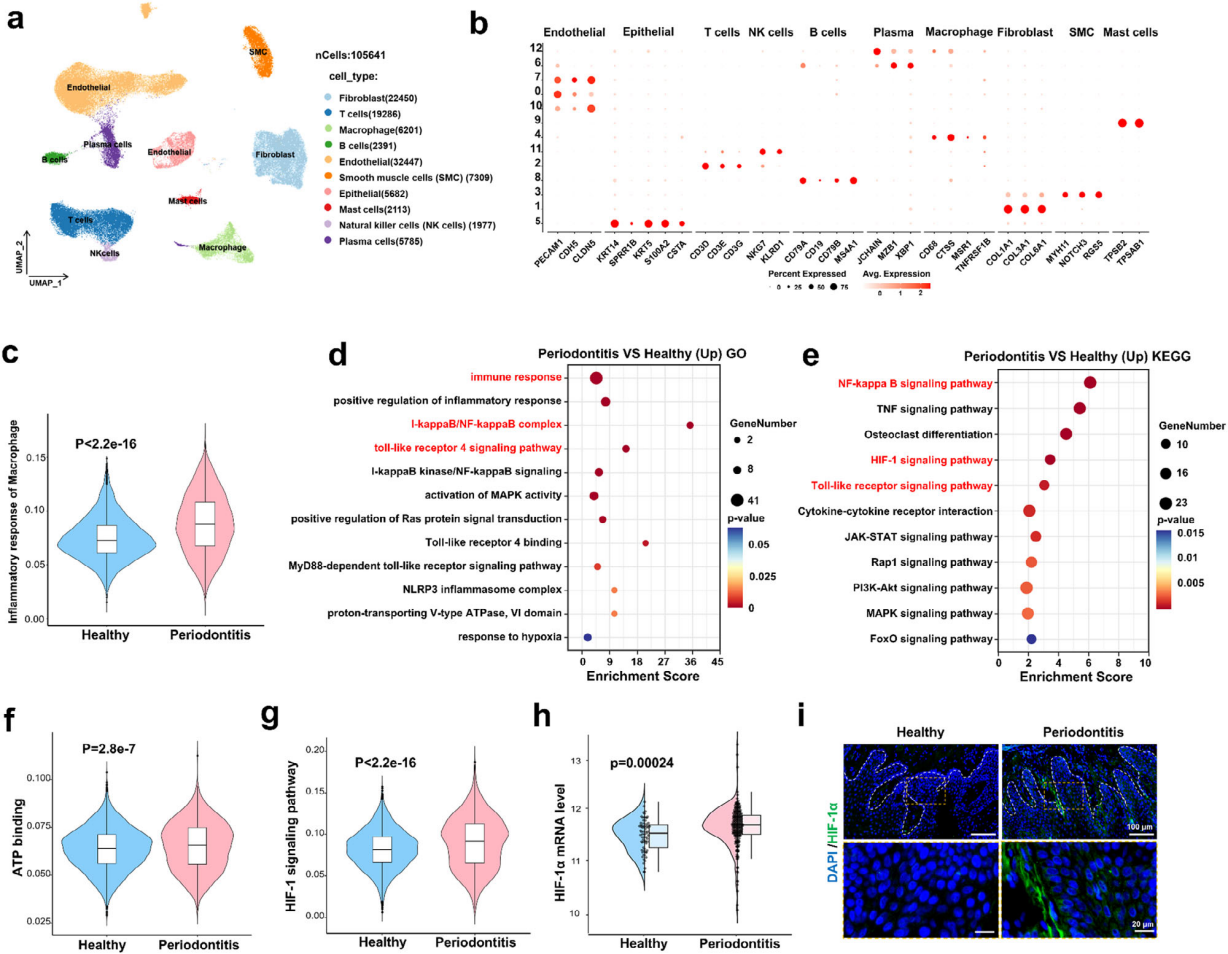
Regulatory mechanisms of macrophages in periodontitis patients revealed by scRNA‐seq analysis. a) UMAP visualization of periodontal tissue‐derived cell clusters from healthy controls and periodontitis patients (data source: GEO accession GSE164241). b) Dot plot depicting canonical marker gene expression across identified clusters. c) Upregulation of inflammatory response pathways in macrophages derived from periodontitis patients. GO (d) and KEGG (e) enrichment analyses highlighting key regulatory, metabolic, and inflammatory pathways in macrophages isolated from periodontitis tissues. f) Significant enrichment of the ATP‐binding pathway in macrophages from periodontitis patients. g) Elevated HIF‐1 signaling pathway activity was identified in macrophages from periodontitis tissues. h) Tissue microarray quantification demonstrating significantly increased HIF‐1α mRNA expression in periodontitis patients compared with healthy controls. i) Immunofluorescence staining illustrating markedly enhanced HIF‐1α protein expression in gingival tissues from periodontitis patients versus healthy controls.

To investigate the immunoregulatory mechanism of macrophages in the progression of periodontitis, we analyzed publicly available single‐cell RNA sequencing (scRNA‐seq) data from healthy individuals and periodontitis patients.^[^ ^25^ ^]^ We analyzed the scRNA‐seq data of human periodontitis samples and annotated ten primary cell types based on canonical marker genes, including fibroblast, T cells, macrophage, B cells, Endothelial, smooth muscle cells, Epithelial, Mast cells, Natural killer cells, and Plasma cells (**Figure** **1**a,b). A comparative analysis of macrophage inflammatory responses revealed significantly elevated inflammatory activity in periodontitis patients compared to healthy controls (Figure 1c). To gain deeper insights, we performed Gene Ontology (GO) and Kyoto Encyclopedia of Genes and Genomes (KEGG) enrichment analyses of differentially expressed genes. These analyses demonstrated substantial upregulation of inflammation‐related pathways in macrophages from periodontitis patients, including NF‐κB, HIF‐1, and Toll‐like receptor signaling pathways (Figure 1d,e). The HIF‐1 pathway, closely linked to the pro‐inflammatory M1 macrophages, was markedly upregulated (Figure 1e,g). Macrophage polarization and inflammatory responses, pivotal components of the immune microenvironment, are intricately dependent on metabolic processes, particularly glycolysis and ATP metabolism.^[^ ^26^ ^]^ Figure 1e,f illustrated pronounced metabolic reprogramming in macrophages from periodontitis patients, characterized by upregulated ATP‐binding pathway.

LPS stimulation activates the Akt/mTOR pathway, enhancing glucose uptake and inducing HIF‐1α expression. These cascades stimulate the glycolytic pathway, leading to elevated transcription and secretion of pro‐inflammatory cytokines.^[^ ^27^ ^]^ Tissue microarray analysis revealed a significant increase in HIF‐1α expression in gingival tissues from periodontitis patients compared to healthy controls (Figure 1h). scRNA‐seq data further confirmed HIF‐1α upregulation in macrophages (Figure S1a, Supporting Information). Under inflammatory conditions, HIF‐1α is rapidly upregulated, driving glycolytic flux and shifting macrophage polarization toward the pro‐inflammatory M1 phenotype.^[^ ^28^ ^]^ Additional analysis of macrophage polarization markers demonstrated that pro‐inflammatory genes associated with the M1 phenotype, such as HIF‐1α, NOD‐like receptor thermal protein domain‐associated protein 3 (NLRP3), and IL‐1β, were significantly elevated in periodontitis‐associated macrophages (Figure S1a—c, Supporting Information). In contrast, anti‐inflammatory genes associated with the M2 phenotype, such as IL‐10RA, Interferon regulatory factor 4 (IRF4) were significantly downregulated (Figure S1d–e, Supporting Information). Immunofluorescence staining of periodontal tissues confirmed elevated HIF‐1α expression in periodontitis patients compared to healthy controls (Figure 1i). These findings underscore the pivotal role of macrophages in the progression of periodontitis, highlighting a distinct polarization shift toward the pro‐inflammatory M1 phenotype. This polarization was characterized by enhanced glycolysis and ATP metabolism, processes partially regulated by the HIF‐1 signaling pathway, emphasizing HIF‐1α as a key regulator in the metabolic reprogramming of macrophages in periodontitis.

### 2.2 Preparation and Characterization of GNPs, HA‐Cu, and HIPPE

**Figure 2:**
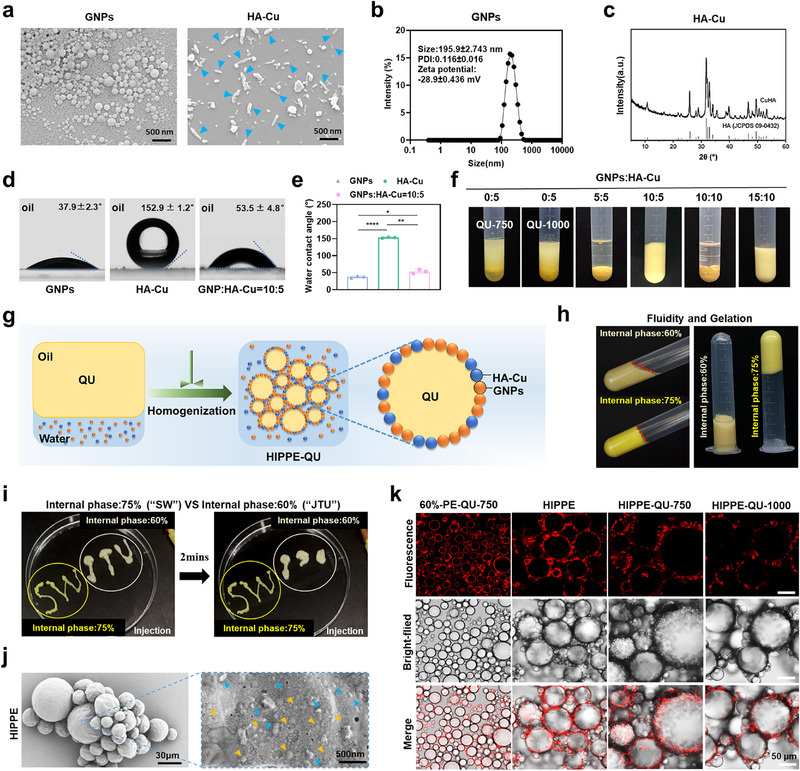
Characterization of GNPs, HA‐Cu, and HIPPE. a) SEM images of GNPs and HA‐Cu. b) Size distribution and zeta‐potential of the synthesized GNPs, measured by dynamic light scattering (DLS). c) XRD patterns of HA‐Cu. d, e) Three‐phase contact angles of GNPs, HA‐Cu, and GNPs: HA‐Cu mixtures in MCT oil(d); corresponding quantification (e). f) Stabilization of oil‐in‐water HIPPE‐QU using different ratios of GNPs and HA‐Cu nanoparticles. g) Schematic illustration of the preparation process of HIPPE‐QU. h) Characterization of fluidity in Pickering emulsions with 60% and 75% internal oil phases. i) Injectability and gel‐like properties of Pickering emulsions with 60% and 75% internal oil phases. j) SEM images of the droplets and their interfacial structure of the HIPPE. Yellow arrows indicate GNPs, and blue arrows indicate HA‐Cu. k) CLSM images of 60%‐PE‐QU‐750, HIPPE, HIPPE‐QU‐750, and HIPPE‐QU‐1000 (HIPPE‐QU‐750 and HIPPE‐QU‐1000 indicate that the QU loading in the oil phase is 750 and 1000 µg mL^−1^, respectively). Data are expressed as mean ± standard deviation (s.d.) (n = 3); * *p* < 0.05; ** *p* < 0.01; **** *p* < 0.0001.

This study utilized HIPPE as a delivery platform, leveraging its gel‐like viscosity and excellent flowability to effectively deliver QU to periodontal pockets while filling tissue gaps. To endow the HIPPE with antibacterial properties and promote tissue regeneration, a co‐stabilizer, HA‐Cu, was synthesized using a hydrothermal method with CuSO_4_ and Ca(OH)_2_ at a (Ca + Cu)/P molar ratio of 1.67, followed by aging, washing, drying, and calcination.^[^ ^29^ ^]^ Scanning electron microscopy (SEM) images revealed that GNPs exhibited a uniform spherical morphology, while HA‐Cu displayed a rod‐like nanostructure, as indicated by the arrows (**Figure** **2**a). The size distribution analysis indicated that GNPs had an average diameter of ≈195.9 nm and a zeta potential of −28.9 mV (Figure 2b), suggesting excellent physical stability due to their high negative charge around 30 mV. This significant negative charge generated strong electrostatic repulsion among nanoparticle‐coated droplets, enhancing Pickering emulsion stability by preventing coalescence, creaming, and Ostwald ripening.^[^ ^30^ ^]^ X‐ray diffraction (XRD) patterns showed typical diffraction peaks corresponding to HA, with no significant copper peaks detected, likely due to low copper concentration (Figure 2c). GNPs exhibited a contact angle of 37.9 ± 2.3°, confirming their hydrophilic properties and suitability for O/W emulsion stabilization, while HA‐Cu showed a hydrophobic contact angle of 152.9 ± 1.2°. Combining GNPs and HA‐Cu adjusted the wettability of the mixed nanoparticles to be 53.5 ± 4.8°, achieving an optimal contact angle approaching an intermediate level (≈90°), which is an ideal value for stabilizing Pickering emulsion (Figure 2d,e).^[^ ^31^ ^]^

To determine the optimal conditions for stable Pickering emulsions using GNPs and HA‐Cu, we performed high‐shear emulsification and one‐step preparation with varying GNPs: HA‐Cu ratios (0:5, 5:5, 10:5, 10:10, and 15:10). The results demonstrated that stable emulsions were achieved at GNPs: HA‐Cu ratios of 10:5 and 15:10 (Figure 2f). This phenomenon suggested that excessive HA‐Cu could inhibit the transformation of GNPs to anchor on the interface during emulsification, leading to macrophase separation. Based on efficiency and stability, a GNPs: HA‐Cu ratio of 10:5 was selected for further HIPPE preparation. QU was dissolved in the oil phase, while nanoparticles (HA‐Cu and GNPs) were dispersed in the aqueous phase. An oil‐in‐water HIPPE‐QU was then prepared by a one‐step homogenization method. In this system, the nanoparticles self‐assemble at the oil–water interface as stabilizers, enabling close packing of droplets and imparting a gel‐like 3D structure and excellent stability (Figure 2g). As shown in Figure 2h, the resultant HIPPE exhibited gel‐like properties, retaining its shape even when inverted. Conversely, a Pickering emulsion with a medium internal phase (60%) displayed liquid‐like properties. Both 60% and 75% internal phase Pickering emulsions could be easily loaded into syringes and extruded through a 0.45 × 16 mm needle to form “SWJTU” letters without blockage (Figure 2i; Movie S1, Supporting Information). The HIPPE maintained its letter shapes without aggregation (Figure 2i), whereas Pickering emulsion with 60% internal phase exhibited completely different rheological properties with full fluidity after extrusion for two minutes. To evaluate the clinical potential of HIPPE‐QU, we assessed its stability under various conditions. At 4 °C and 37 °C, both HIPPE and HIPPE‐QU maintained stability for over three months, indicating exceptional biphasic system stability (Figure S2, Supporting Information).

SEM images presented the microstructure of the interface of emulsion droplets, with both GNPs and HA‐Cu nanoparticles anchoring around the droplet interfaces, confirming their co‐stabilization effect (Figure 2j). Owing to the autofluorescent properties, GNPs emitted red fluorescence under 543 nm laser excitation,^[^ ^32^ ^]^ and the confocal laser scanning microscopy (CLSM) revealed that GNPs were irreversibly adsorbed at the oil–water interface, forming a thick interfacial layer that prevented droplet coalescence. With a 75% internal phase (HIPPE), droplets were tightly packed, forming multiple contact points that yielded a gel‐like network, whereas the emulsion droplets remained individually dispersed with a 60% internal phase (60%‐PE) (Figure 2k). This difference arises from the increased droplet packing density at higher internal phase volumes, where deformed droplets become closely associated, forming a percolated network with strong inter‐droplet interactions that restrict flow and confer solid‐like rheological behavior.^[^ ^33^ ^]^ Introducing QU altered the HIPPE microstructure, significantly enlarging droplet size and broadening their size distribution (Figure S3a–d, Supporting Information). Additionally, the presence of QU modified the interfacial structure of emulsion droplets, as evidenced by the disappearing fluorescence signal around the interfaces. This phenomenon was enhanced with the increase of QU, suggesting the appearance of some new substitute components on the interfaces.

### 2.3 The Stabilization Mechanism and Properties of HIPPE‐QU

**Figure 3:**
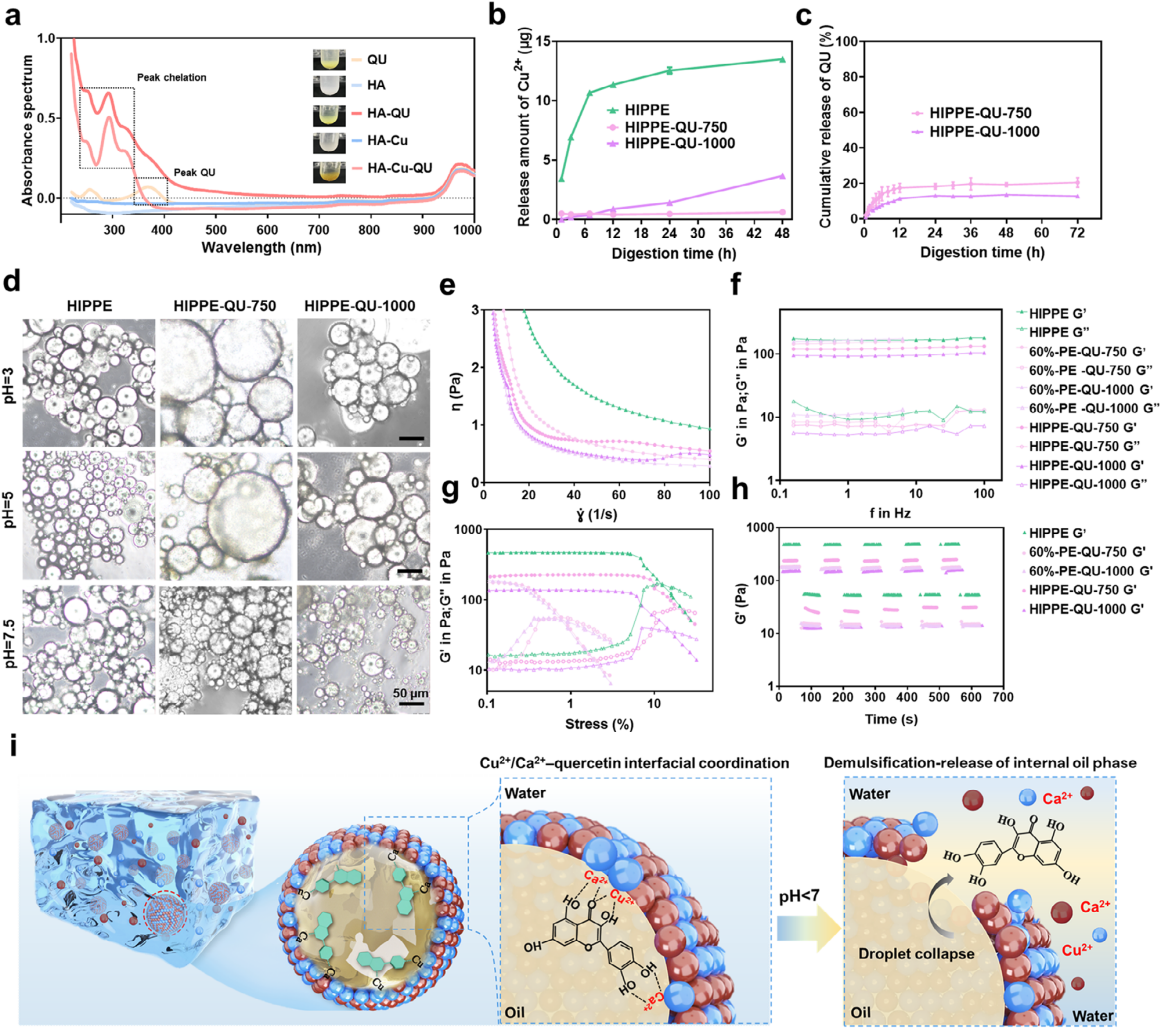
Characterization of HIPPE, HIPPE‐QU‐750, HIPPE‐QU‐1000, and 60%‐PE‐QU‐750. a) UV–vis absorption spectra of QU, HA, HA‐Cu, HA‐QU, and HA‐Cu‐QU in pure water. b) Cu^2+^ release profiles measured by ICP‐MS. c) Cumulative QU release curves from HIPPE with varying QU concentrations (n = 3). d) Microstructural analysis of HIPPE, HIPPE‐QU‐750, HIPPE‐QU‐1000 under pH 3, 5, and 7.5 conditions. e) Shear rate‐dependent viscosity of HIPPE, HIPPE‐QU‐750, HIPPE‐QU‐1000, and 60%‐PE‐QU‐750. f) Frequency sweep analysis of G' and G″ for HIPPE, HIPPE‐QU‐750, HIPPE‐QU‐1000, 60%‐PE‐QU‐750, and 60%‐PE‐QU‐1000 at 1 Hz. g) Strain sweeps the behavior of G′ and G″ for emulsions at 1 Hz. h) Rheological recovery performance of emulsions under alternating strain conditions. i) Schematic illustration of the interface structure and acid‐triggered drug release characteristics of HIPPE‐QU.

To investigate the stabilization mechanism of HIPPE containing mixed nanoparticles and bioactive compounds in the internal phase, we analyzed the interfacial behavior of the material involved. The emission spectra of QU, HA‐QU, and HA‐Cu‐QU were analyzed over the 200–1000 nm range. The results revealed a characteristic emission peak for QU at ≈370 nm, while peaks for HA‐QU and HA‐Cu‐QU exhibited significant shifts and increased intensities, accompanied by a color change from light yellow to dark yellow (**Figure** **3**a). This observation confirmed the successful chelation of QU with Cu^2^⁺ and Ca^2^⁺, consistent with previous findings.^[^ ^34^ ^]^ We speculated that this chelation effect facilitated the migration of HA‐Cu to the oil‐water interface, as observed via CLSM, where a substantial proportion of GNPs was replaced at the interface (Figure 2k). Inductively coupled plasma mass spectrometry (ICP‐MS) analysis of Cu^2^⁺ release from HIPPE with varying QU content demonstrated distinct release profiles, with Cu^2^⁺ release from HIPPE‐QU being significantly reduced compared to HIPPE formulations lacking QU (Figure 3b). Within 48 h, the incorporation of QU reduced Cu^2^⁺ release by more than tenfold (Figure 3b). This suppression in Cu^2^⁺ release likely resulted from the interfacial chelation‐engineered structure formed between QU and Cu^2^⁺, which significantly fixed the HA‐Cu around the droplet interfaces and thus enabled controlled and long‐term delivery of Cu^2^⁺. Increasing QU content intensified the chelation effect but compromised emulsion stability due to the reduced interfacial density. Consequently, droplet structures broke down, leading to a slight increase in Cu^2^⁺ release in HIPPE‐QU‐1000 compared to HIPPE‐QU‐750 after 24 h of incubation. QU release profiles revealed a rapid increase followed by plateauing after 14 h (Figure 3c). The cumulative release of QU in HIPPE‐QU‐750 was higher than that in HIPPE‐QU‐1000, likely due to enhanced chelation effects in the latter, which slowed the release of QU.

The emulsion stability under different pH conditions was investigated. At pH 3, 5, and 7.5, GNPs and HA‐Cu synergistically preserved HIPPE stability over a defined period (Figure 3d). However, QU incorporation significantly affected stability under acidic conditions. At pH 3 and 5, droplets in HIPPE‐QU‐750 and HIPPE‐QU‐1000 were significantly larger than those in HIPPE. Acidic conditions altered nanoparticle surface charges, while chelation of QU with Cu^2^⁺ and Ca^2^⁺ reduced interfacial density, all promoted droplet coalescence. This pH sensitivity indicated that HIPPE‐QU facilitated rapid QU release under acidic periodontal conditions, which were typically associated with inflammation and bacterial infection.

The mechanical properties of 60%‐PE and HIPPE were comprehensively studied and compared by rheological investigation. Viscosity measurements demonstrated significant shear‐thinning behavior across all the emulsions, indicating reduced viscosity with increasing shear rate (Figure 3e). HIPPE droplets, typically highly flocculated and polyhedral due to their large internal phase volume fraction, behave as elastic solids under low shear strain because of increased inter‐droplet friction, but exhibit shear‐thinning behavior once the yield stress is exceeded, as droplets reorganize and deform along the shear direction.^[^ ^35^ ^]^ This property facilitates emulsion delivery into irregular periodontal pockets. Incorporating QU substantially decreased emulsion viscosity, suggesting the reduced interfacial viscoelasticity caused by chelation effects. Frequency sweep analysis indicated that the storage modulus (G′) remained higher than the loss modulus (G″) across the angular frequency range, confirming elastic gel‐like behavior with a steady plateau region (Figure 3f). Amplitude sweep tests revealed that G′ exceeded G″ at low strains due to the interfacial tension resisting droplet deformation.^[^ ^36^ ^]^ However, G′ gradually declined and fell below G″ when strain exceeded the yield strain, signifying structural rearrangement or droplet flow (Figure 3g). HIPPE resisted gel structure collapse up to ≈10% sweep strain, whereas 60%‐PE collapsed at ≈1%. This disparity is attributed to the densely packed droplets in HIPPE,^[^ ^37^ ^]^ which could resist being deformed. Rheological recovery analysis under alternating strain conditions demonstrated excellent self‐healing properties. At 1% applied strain, G' remained higher than G″. When strain increased to 30%, G′ declined sharply, falling below G″, indicating the breakdown between the densely packed droplets in HIPPE. Upon returning strain to 1%, the microstructure reorganized, and G′ and G″ rapidly recovered to their original values, even after five repeated cycles (Figure 3h). These findings confirmed that HIPPE‐QU exhibited a robust “deform‐recover‐deform” oscillatory cycle, highlighting its effective self‐healing capability. At the interface, QU chelated Cu^2^⁺ and Ca^2^⁺ to form stable interfacial complexes. Under acidic conditions (pH < 7), the stability of these interfacial complexes decreased, leading to the collapse of the oil droplets and the release of QU. Subsequently, Cu^2^⁺ and Ca^2^⁺ were also released from the interface, enabling stepwise and precise delivery of multiple components. This sequential release not only avoided the burst‐release toxicity of Cu^2^⁺ but also prolonged the effect of Ca^2^⁺ (Figure 3i).

### 2.4 Antibacterial Activities and Biocompatibility of HIPPE‐QU in Vitro

**Figure 4:**
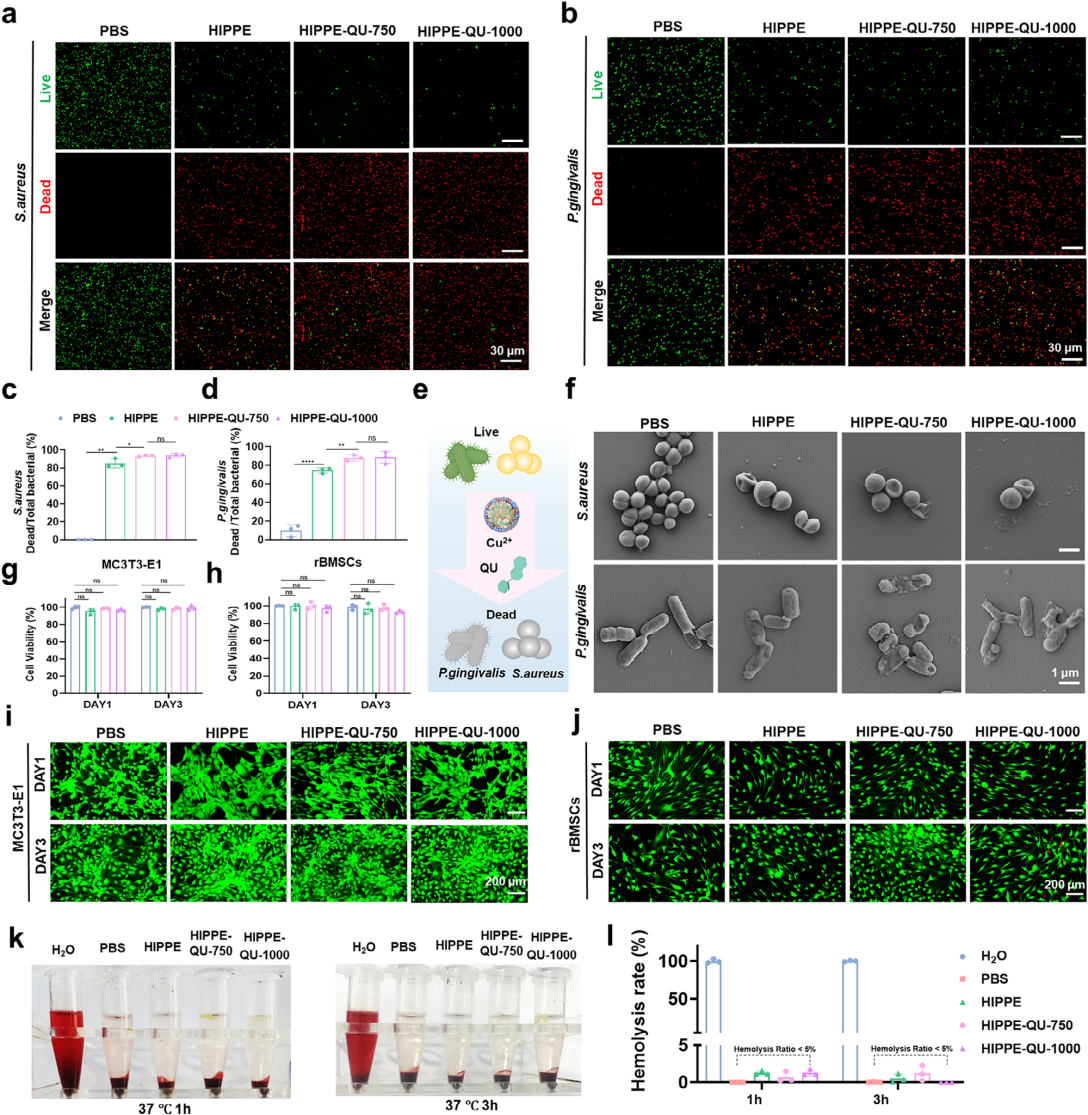
In vitro antibacterial and biocompatibility evaluation of HIPPE‐QU. a,b) Representative fluorescence live/dead staining images of *S. aureus* (a) and *P. gingivalis*. (b) treated with HIPPE and HIPPE‐QU for 24 h. Live bacteria are shown in green, and dead bacteria are shown in red. c,d) Quantitative analysis of live/dead staining for *S. aureus* (c) and *P. gingivalis*(d). e) Schematic illustration of the antibacterial effects of HIPPE‐QU. (**f**) Representative SEM images of *S. aureus* and *P. gingivalis* after 24‐h treatment with HIPPE and HIPPE‐QU g,h) MTT assay results of MC3T3‐E1 (g) and rBMSCs (h) cultured with HIPPE, HIPPE‐QU for 1 and 3 days. i,j) Representative fluorescence live/dead staining images of MC3T3‐E1 (i) and rBMSCs (j) after 1 and 3 days of culture with HIPPE, HIPPE‐QU. Live cells are shown in green, and dead cells are shown in red. k,l)Representative images (k) and quantification of hemolysis rates (%)(l) of red blood cell suspensions after incubation with H_2_O, PBS, HIPPE, HIPPE‐QU‐750, and HIPPE‐QU‐1000 at 37 °C for 1 h and 3 h. Data are expressed as mean ± standard deviation (s.d.), with n = 3; * *p* < 0.05; ** *p* < 0.01; **** *p* < 0.0001; ns, not significant.

The imbalance of the periodontal microbiome is considered a key factor in the pathogenesis of periodontal disease.^[^ ^38^ ^]^ To comprehensively evaluate the broad‐spectrum antibacterial performance of the HIPPE‐QU system, we selected both Gram‐negative and Gram‐positive bacteria relevant to periodontal infections. *P. gingivalis* was chosen as a representative Gram‐negative keystone pathogen, as it plays a dominant role in immune disruption and alveolar bone resorption during periodontitis progression.^[^ ^39^ ^]^ In contrast, *S. aureus* was included as a Gram‐positive opportunistic pathogen, particularly known for its involvement in biofilm‐associated infections and secondary periodontal complications.^[^ ^40^ ^]^ We assessed the antibacterial activity of HIPPE and HIPPE‐QU against *S. aureus* and *P. gingivalis*. Live/dead staining revealed significantly increased red fluorescence (PI staining) in bacteria treated with HIPPE and HIPPE‐QU compared to the PBS group (**Figure** **4**a,b). Quantitative analysis demonstrated that the bactericidal rates against *S. aureus* exceeded 90% for both HIPPE‐QU‐750 and HIPPE‐QU‐1000, significantly higher than the 84.94% observed in the HIPPE group (Figure 4c). In parallel, inhibition rates against *P. gingivalis* were significantly improved, reaching approximately 88% for HIPPE‐QU‐750 and HIPPE‐QU‐1000, compared to 74.5% in the HIPPE group (Figure 4d). HIPPE exhibited strong antibacterial activity, which was primarily attributed to the release of Cu^2^⁺. Incorporation of QU into HIPPE (HIPPE‐QU) further enhanced the bactericidal activity, likely due to a synergistic antibacterial effect arising from the combined release of QU and Cu^2^⁺ (Figure 4e). The SEM analysis showed that *S. aureus* and *P. gingivalis* in the PBS group exhibited normal spherical and rod‐shaped structures, respectively, with intact biofilms. In contrast, after treatment with HIPPE and HIPPE‐QU, the bacterial surfaces appeared rough and collapsed, with numerous perforations (Figure 4f). Our results demonstrated that HIPPE and HIPPE‐QU exerted bactericidal effects by disrupting the cell membrane structures of *S. aureus* and *P. gingivalis*, ultimately leading to bacterial membrane rupture and cell death.

Biocompatibility is fundamental to the clinical application of biomaterials, as it ensures cell viability and proliferation.^[^ ^41^ ^]^ Live/dead staining revealed no significant dead cells in pre‐osteoblast cells (MC3T3‐E1) and rBMSCs treated with HIPPE or HIPPE‐QU, indicating that both HIPPE and HIPPE‐QU promoted excellent biocompatibility (Figure 4i,j). For rBMSCs, the HIPPE‐QU‐750 exhibited the highest cell density after 72 h, whereas the HIPPE‐QU‐1000 group displayed some dead cells (Figure 4j). MTT assay results were consistent with these observations. After 24 and 72 h of co‐culture with MC3T3‐E1 and rBMSCs, cell viability in all groups exceeded 90%, further confirming the excellent biocompatibility of both HIPPE and HIPPE‐QU (Figure 4g,h). Moreover, the hemolysis assay showed that after incubation with red blood cells at 37 °C for 1 and 3 h, the hemolysis rates of the HIPPE, HIPPE‐QU‐750, and HIPPE‐QU‐1000 groups were all below the ISO standard threshold (5%), indicating no hemolysis occurred (Figure 4k,l). These results demonstrated that the system possessed excellent hemocompatibility.

### 2.5 Evaluation of HIPPE‐QU‐Mediated ROS Scavenging, Mitochondrial Functional Recovery, and Macrophage Polarization

**Figure 5:**
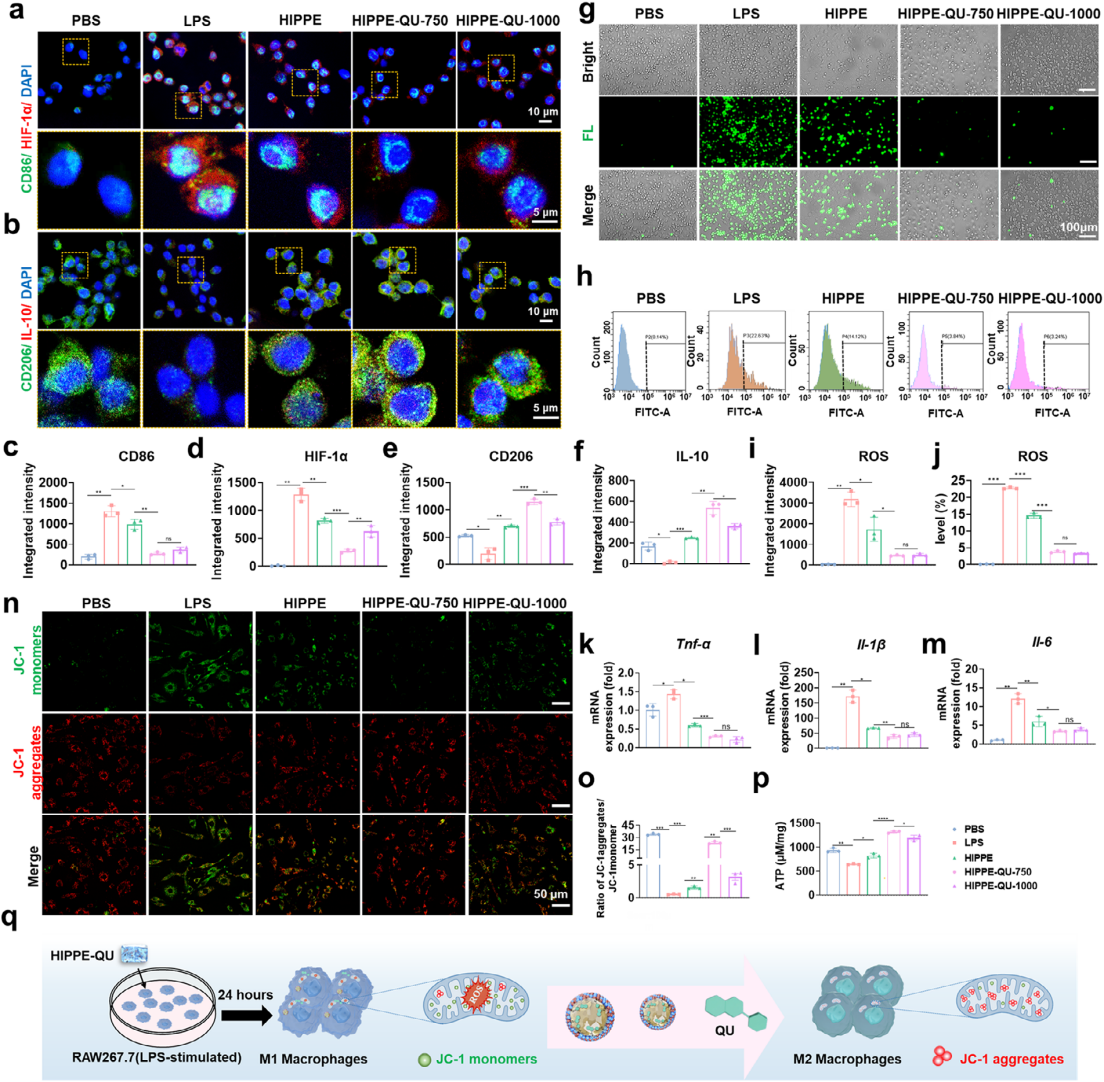
In vitro ROS scavenging, mitochondrial reprogramming, and macrophage polarization by HIPPE‐QU. a, b) RAW264.7 macrophages stimulated with LPS (100 ngmL^−1^, 24 h) ± HIPPE‐QU; immunofluorescence images of M1 markers CD86 (green) and HIF‐1α (red) (a) and M2 markers CD206 (green) and IL‐10 (red) (b). c–f) Quantification of fluorescence intensity for CD86 (c), HIF‐1α (d), CD206 (e), and IL‐10 (f). g,i) Fluorescence images of intracellular ROS in LPS‐stimulated macrophages ± HIPPE‐QU (g); corresponding quantification (i). h,j) Flow cytometry of ROS levels in macrophages after 24 h LPS ± HIPPE‐QU (h)corresponding quantification (j). k–m) mRNA levels of pro‐inflammatory cytokines *Tnf‐α* (k), *Il‐1β* (l), and *Il‐6* (m). n, o) JC‐1 staining for MMP (n); quantification of the red (aggregate) green (monomer) ratio (o). p) ATP levels in RAW264.7 macrophages after 24 h LPS ± HIPPE‐QU. q) Schematic illustration of in vitro ROS scavenging, mitochondrial reprogramming, and macrophage polarization by HIPPE‐QU. Data are presented as mean ± standard deviation (s.d.) (n = 3); *p < 0.05; **p < 0.01; ***p < 0.001; ****p < 0.0001; ns, not significant.

To investigate the regulatory effects of HIPPE‐QU on macrophage polarization, RAW264.7 macrophages were polarized toward the M1 phenotype using LPS and then co‐cultured with HIPPE, HIPPE‐QU‐750, and HIPPE‐QU‐1000. CD86 and HIF‐1α were utilized as markers of M1 macrophages. Immunofluorescence confirmed successful M1 polarization in LPS‐treated cells, evidenced by markedly increased CD86 and HIF‐1α coupled with decreased CD206 and IL‐10 (**Figure** **5**a–f). Consistently, qRT‐PCR showed that LPS treatment dramatically elevated *Tnf‐α*, *Il‐1β*, and *Il‐6* mRNA levels, consistent with an M1 inflammatory phenotype (Figure 5k–m). Upon co‐culture with HIPPE or HIPPE‐QU, these LPS‐induced M1 markers were significantly attenuated: both treatments reduced CD86 and HIF‐1α fluorescence while enhancing the M2 markers CD206 and IL‐10 (Figure 5a–f). HIPPE‐QU was more efficacious than HIPPE, with the HIPPE‐QU‐750 group displaying the most pronounced effects (Figure 5a–f). qRT‐PCR corroborated these findings by showing that both HIPPE and HIPPE‐QU significantly downregulated the mRNA levels of pro‐inflammatory cytokines (Figure 5k,m). The superior anti‐inflammatory effect of HIPPE‐QU suggested a synergistic inhibitory action mediated by the combined release of Cu^2^⁺ and QU. However, no significant differences were observed between HIPPE‐QU‐750 and HIPPE‐QU‐1000, likely due to the sustained‐release dynamics at the 24 h’ time point. In this system, the chelation between Cu^2^⁺ and QU at the oil–water interface formed stable coordination complexes, which modulated the release kinetics of both QU and Cu^2^⁺.

To assess whether HIPPE‐QU mitigated oxidative stress and redirected polarization toward the M2 phenotype, RAW264.7 macrophages were treated with LPS to simulate the inflammatory oxidative stress associated with periodontitis. ROS levels were quantified using DCFH‐DA probes, which showed a significant increase in fluorescence intensity in the LPS group compared to the PBS control, confirming successful oxidative stress induction. Both HIPPE and HIPPE‐QU effectively attenuated ROS levels, with HIPPE‐QU demonstrating superior scavenging activity (Figure 5g,i). Flow cytometry further confirmed that HIPPE‐QU reduced LPS‐induced ROS production by approximately 83%, emphasizing its potent antioxidant effects (Figure 5h,j).

Mitochondria, as central regulators of macrophage metabolic reprogramming, are particularly vulnerable to excessive ROS, which can cause irreversible structural damage and dysfunction.^[^ ^42^ ^]^ QU has been shown to modulate macrophage inflammatory responses by stabilizing mitochondrial membrane potential (MMP).^[^ ^43^ ^]^ To investigate the protective effects of HIPPE‐QU on mitochondrial function, JC‐1 probes were used to assess MMP in RAW264.7 macrophages under different treatments. In LPS‐treated cells, green fluorescence (indicative of JC‐1 monomers) predominated, reflecting a significant reduction in MMP. Treatment with HIPPE‐QU significantly restored MMP, shifting fluorescence toward red (indicative of JC‐1 aggregates), thereby reflecting improved mitochondrial integrity (Figure 5n,o). Remarkably, the HIPPE‐QU‐750 group achieved MMP levels comparable to the PBS control, demonstrating its robust mitochondrial protective effects. Moreover, LPS treatment substantially reduced ATP production,^[^ ^28^ ^]^ reflecting impaired mitochondrial function. HIPPE‐QU treatment significantly restored ATP levels, indicating the preservation of mitochondrial bioenergetics (Figure 5p). HIPPE‐QU significantly enhanced anti‐inflammatory effects by promoting macrophage M2 polarization, improving mitochondrial function, and efficiently scavenging ROS to reduce oxidative stress and tissue damage. Additionally, QU's chelation with Cu^2^⁺ in the Pickering emulsion enabled its sustained release, prolonging anti‐inflammatory action (Figure 5q).

### 2.6 HIPPE‐QU Modulates Macrophage Polarization by Regulating Metabolic Reprogramming Through the HIF‐1 Signaling Pathway

**Figure 6:**
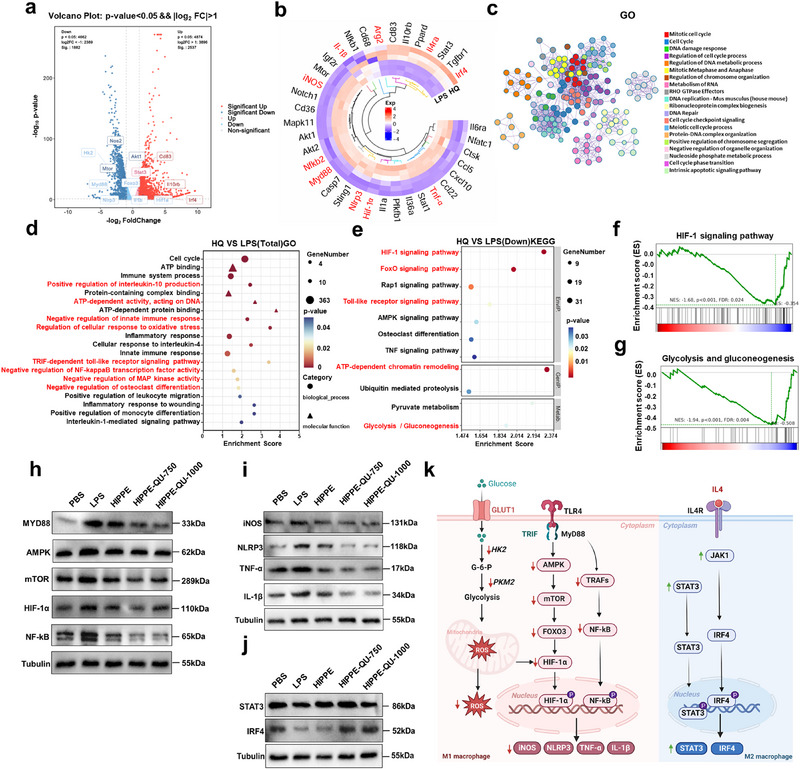
Mechanistic insights into HIPPE‐QU‐750 (HQ)‐mediated macrophage polarization and inflammatory metabolic reprogramming. a) Volcano plot of differentially expressed genes (DEGs) between the HQ and LPS groups. b) Circular heatmap of key DEGs associated with macrophage polarization and inflammatory metabolism in the HQ treatment group. c,d) GO enrichment analysis of DEGs highlighting significantly enriched biological processes (c) and molecular functions (d) modulated by HQ. e) KEGG pathway enrichment analysis f, g) GSEA analysis showing HQ‐mediated suppression of the HIF‐1 signaling (f) and glycolysis/ gluconeogenesis (g) pathways. h) Western blot analysis of key signaling proteins in the HIF‐1 pathways. i) Western blot analysis of periodontal inflammation‐related proteins. j) Western blot for STAT3 and IRF4. k) Schematic illustration of the mechanism by which HQ regulates macrophage polarization and inflammatory metabolism. HQ releases Cu^2^⁺ and QU, providing antioxidant effects that suppress LPS‐induced glycolysis in M1 macrophages, restore MMP, and enhance ATP production thereby inhibiting ROS generation and M1 macrophage polarization. HQ also downregulates Toll‐like receptor, AMPK, and HIF‐1 signaling pathways, reducing pro‐inflammatory mediator (iNOS, NLRP3, TNF‐α, IL‐1β), while upregulating STAT3 and IRF4 expression to promote M2 macrophage polarization with increased M2 markers (IL4, Arg2, CD206, and IL10) and enhanced anti‐inflammatory cytokine secretion.

To elucidate the molecular mechanisms underlying HIPPE‐QU‐mediated regulation of macrophage polarization within an inflammatory immune microenvironment, transcriptome sequencing was performed on RAW264.7 macrophages stimulated with LPS for 24 h and treated with HIPPE‐QU. Volcano plot analysis revealed significant differential gene expression, with 2537 genes upregulated and 1882 genes downregulated in the HIPPE‐QU group compared to the LPS group (**Figure** **6**a). Among these differentially expressed genes, key M1 phenotype‐associated genes, such as *iNos*, *Nlrp3*, *Tnf‐α*, *Il‐1β*, and *Hif‐1α* were markedly downregulated, while M2 phenotype‐associated genes, including *Arg2*, *Stat3*, and *Il4ra* were significantly upregulated (Figure 6b, Figure S4a,b, Supporting Information). GO enrichment analysis demonstrated that HIPPE‐QU regulated processes related to immune system modulation, inflammatory responses, oxidative stress, ATP‐dependent activity, Toll‐like receptor signaling, IL‐10 regulation, NF‐κB transcriptional activity, MAP kinase activity, and osteoclast differentiation (Figure 6c,d). Additionally, KEGG pathway analysis revealed substantial suppression of glycolysis/gluconeogenesis pathways and ATP‐dependent chromatin remodeling, as well as metabolic regulatory pathways such as HIF‐1, FoxO, and Toll‐like receptor signaling (Figure 6e). Among these, the HIF‐1 signaling pathway exhibited the strongest enrichment (Figure 6f). Building on previous findings that HIF‐1 signaling played a pivotal role in the metabolic reprogramming of macrophages in periodontitis (Figure 1c–i), GSEA and heatmap analyses further demonstrated significant downregulation of glycolysis‐related genes, including hexokinase *(HK)1/2*, pyruvate kinase M 2 (*Pkm*2), and glucose transporter 1 (*Glut1*) in the HIPPE‐QU‐treated group (Figure 6e,g; Figure S4d, Supporting Information). HIPPE‐QU significantly suppressed LPS‐induced glycolysis through the synergistic release of QU and Cu^2^⁺ (Figure 6e,g).

We further identified key factors involved in HIPPE‐QU‐mediated metabolic reprogramming and immune regulation in M1 macrophages. HIPPE‐QU significantly downregulated glycolysis‐related metabolic factors and signaling molecules, including HK2, PKM, MAPK, mTOR, FOXO3, HIF‐1α, and NF‐κB, as well as pro‐inflammatory mediators such as iNOS, NLRP3, TNF‐α, and IL‐1β (Figure 6b; Figure S4a, f, Supporting Information). In addition, HIPPE‐QU inhibited activation of Toll‐like receptor and HIF‐1 signaling pathways, markedly reducing the expression of key molecules, including MAPK, mTOR, HIF‐1α, iNOS, and NLRP3 (Figure S4f, Supporting Information). Protein‐protein interaction (PPI) network analysis identified central regulatory molecules, including NF‐κB subunits, MYD88, mTOR, and HIF‐1α, as critical mediators of M1 macrophage polarization and immune regulation (Figure S4g, Supporting Information). Western blot analysis further confirmed that HIPPE‐QU effectively suppressed LPS‐induced activation of Toll‐like receptor and HIF‐1 signaling pathways. Key proteins, including MYD88, AMPK, mTOR, HIF‐1α, and NF‐κB, exhibited the most significant downregulation in the HIPPE‐QU‐750 group (Figure 6h). Additionally, critical inflammatory mediators associated with periodontitis, such as iNOS, NLRP3, TNF‐α, and IL‐1β, were significantly modulated (Figure 6i). These findings highlighted that HIPPE‐QU suppressed M1 macrophage polarization and inflammation by targeting Toll‐like receptor and HIF‐1 signaling pathways.

Gene enrichment analysis identified significant upregulation of M2 phenotype‐associated genes, including *Arg2*, *Stat3*, and *Irf4*, in the HIPPE‐QU‐treated group (Figure 6b). Western blot analysis confirmed increased protein expression of M2 polarization markers, including STAT3 and IRF4, providing robust evidence for the efficacy of HIPPE‐QU in promoting M2 macrophage polarization (Figure 6j). In conclusion, HIPPE‐QU regulated glycolytic activity and mitochondrial function by downregulating Toll‐like receptor and HIF‐1 signaling pathways. These mechanisms inhibited M1 macrophage polarization, restored mitochondrial function, and promoted the transition to M2 macrophages (Figure 6k).

### 2.7 Effects of HIPPE‐QU on the Osteogenic Potential of rBMSCs and Gingival Regeneration of HGFs in Vitro Inflammatory Microenvironment

**Figure 7:**
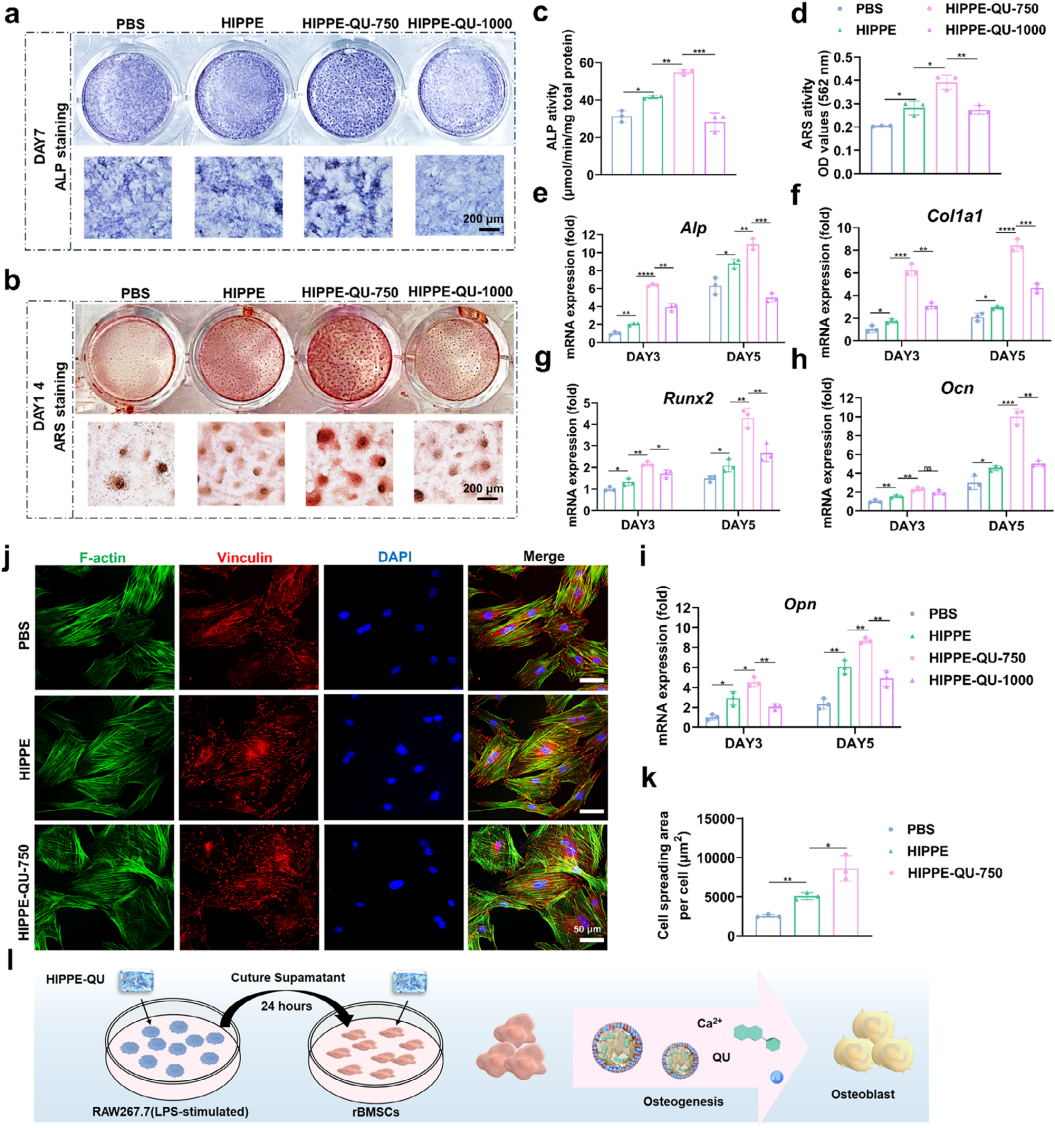
Effects of HIPPE‐QU on the osteogenic potential of rBMSCs and the gingival regeneration of HGFs under inflammatory conditions. a, b) Representative ALP (a) and ARS (b) staining images of rBMSCs cultured with macrophage‐conditioned medium (1:2 ratio with osteogenic induction medium) for 7 and 14 days. c, d) Quantitative analysis of ALP activity (c) and ARS mineralization (d). e–i) qRT‐PCR analysis of osteogenic gene expression, including *Alp* (e), *Col1a1* (f), *Runx2* (g), *Ocn* (h), and *Opn* (i), after 3 and 5 days of co‐culture. (j) Immunofluorescence images of rBMSCs morphology on HIPPE and HIPPE‐QU‐750 surfaces. Actin filaments were labeled with phalloidin (green), nuclei with DAPI (blue), and vinculin (red) highlighted adhesion sites. (k) Quantification of cell spreading areas. (l) Schematic illustration of the osteogenesis‐promoting effects of HIPPE‐QU. Data are expressed as mean ± standard deviation (s.d.) (n = 3). *p < 0.05, **p < 0.01, ***p< 0.001 and ****p < 0.001.

To simulate the periodontal inflammatory microenvironment and evaluate the osteogenic potential of HIPPE‐QU, the conditioned medium from LPS‐stimulated macrophages was collected and co‐cultured with rBMSCs in osteogenic induction medium. Alkaline phosphatase (ALP) staining revealed significantly increased staining intensity in both HIPPE and HIPPE‐QU groups compared to the PBS control, with the HIPPE‐QU‐750 group demonstrating the most pronounced enhancement (**Figure** **7**a). Consistent with these observations, ALP activity assays confirmed that HIPPE‐QU‐750 significantly promoted early osteogenic differentiation under inflammatory conditions after 7 days (Figure 7c). Alizarin Red S (ARS) staining further revealed abundant calcium nodules in the HIPPE and HIPPE‐QU groups, with HIPPE‐QU‐750 achieving the highest quantitative levels of mineralization after 14 days (Figure 7b and d). qRT‐PCR results revealed that both HIPPE and HIPPE‐QU significantly upregulated the expression of osteogenic‐related genes on days 3 and 5 of induction, including *Alp*, *Col1a1*, *Runx2*, *Ocn* and *Opn*, with the HIPPE‐QU‐750 showing the most pronounced increases (Figure 7e–i). The adhesion and spreading of rBMSCs on biomaterial surfaces are critical determinants of successful bone formation.^[^ ^44^ ^]^ Given the superior osteogenic potential of HIPPE‐QU‐750, we focused on comparing this group with HIPPE. After one day of culture, immunofluorescence confocal microscopy of rBMSCs revealed significantly higher fluorescence intensity for vinculin, a key adhesion marker, in HIPPE‐QU‐750 group. Moreover, phalloidin staining of F‐actin demonstrated enhanced spreading and increased cytoskeletal tension, as evidenced by the formation of actin stress fibers in HIPPE‐QU‐750 group compared to other groups (Figure 7j,k; Figure S6, Supporting Information). Overall, it proved that HIPPE‐QU significantly promoted the osteogenic differentiation of rBMSCs under inflammatory conditions (Figure 7l).

To evaluate the effects of HIPPE‐QU on human gingival fibroblasts (HGFs) under inflammatory conditions, LPS‐stimulated macrophages were co‐cultured with HIPPE formulations for 1 day, and the conditioned medium was used to culture HGFs. Key adhesion and migration markers, including Focal Adhesion Kinase (*FAK*), Integrin β1 (*ITGB1*), and Vinculin (*VCL*), along with proliferation and ECM‐related genes, such as Fibronectin (*FN1*) and *COL1A1*, were analyzed using qRT‐PCR. HIPPE‐QU‐750 significantly upregulated the expression of these markers, indicating its capacity to promote soft tissue repair and gingival regeneration under inflammatory conditions (Figure S5a–e, Supporting Information).

### 2.8 HIPPE‐QU‐750 Enhances Osteogenic Differentiation in an Inflammatory Microenvironment via Modulation of Cellular Energy Metabolism and Activation of MAPK Signaling

**Figure 8:**
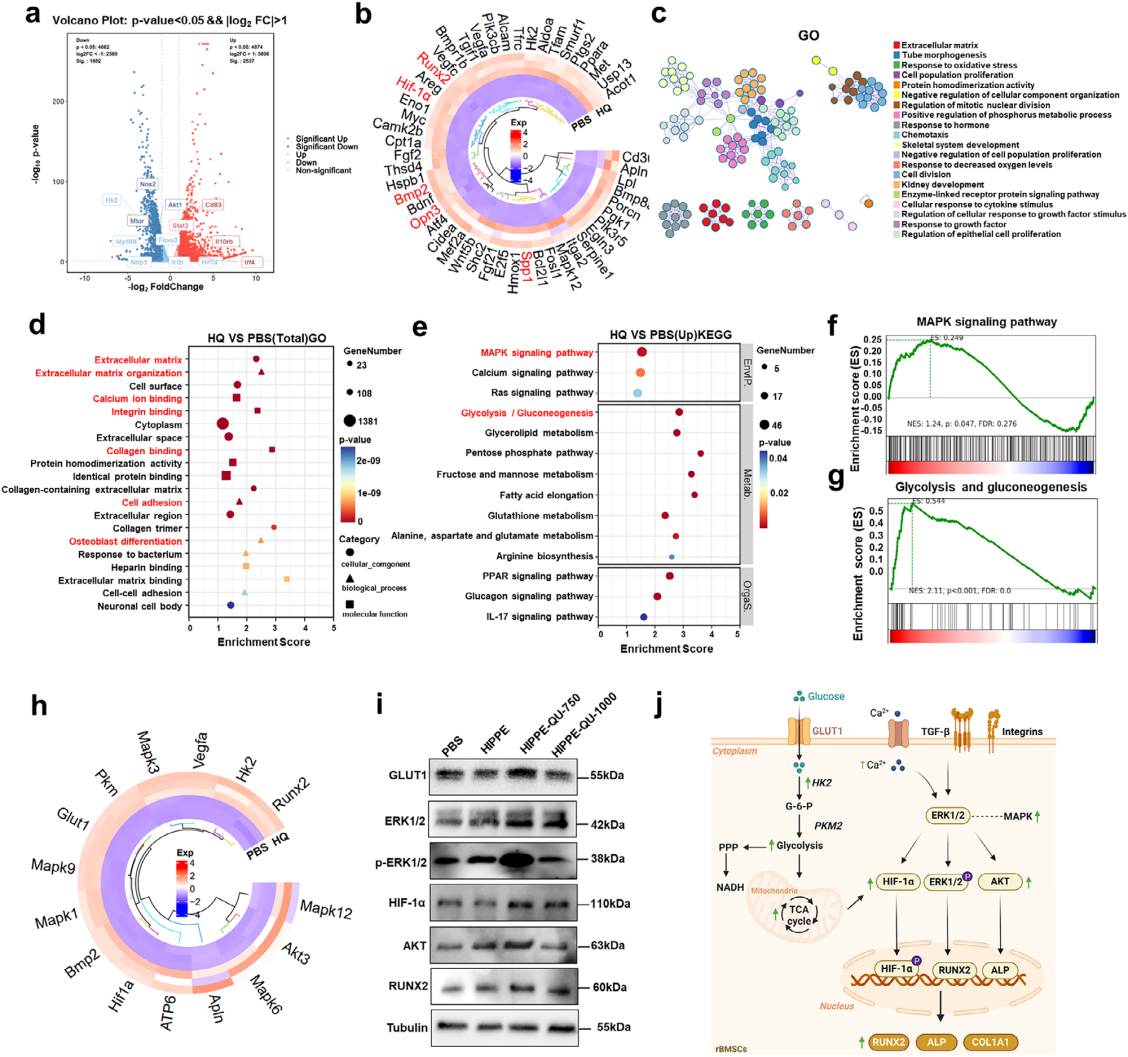
Mechanism of HQ in promoting osteogenic differentiation of rBMSCs. a) Volcano plot of DEGs in HQ group versus PBS group. b) Circular heatmap highlighting HQ‐induced upregulation of key osteogenesis‐related genes. c, d) GO enrichment analysis of DEGs, highlighting significantly enriched cellular components, biological processes, and molecular functions. e) KEGG pathway enrichment analysis showing significantly upregulated signaling pathways and regulatory mechanisms following HQ treatment. f,g) GSEA plots of pathways upregulated by HQ: f) MAPK signaling pathway, and g) glycolysis and gluconeogenesis. h) Circular heatmap showing HQ‐induced upregulation of key MAPK pathway components and osteogenic markers. i) Western blot analysis confirming HQ‐induced activation of MAPK pathways. j) Schematic of the proposed mechanism by which HQ enhances osteogenic differentiation. HQ upregulates glucose metabolism‐related proteins (GLUT1, HK2, PKM2), increasing ATP production via glycolysis and the TCA cycle to meet the energy demands of osteogenesis. HQ also modulates integrin–ECM signaling and elevates intracellular, leading to MAPK activation. Activated MAPK stabilizes HIF‐1α and induces the expression of osteogenic proteins (RUNX2, ALP, COL1A1).

To investigate how HIPPE‐QU‐750 enhanced osteogenic differentiation via macrophage interaction in an inflammatory microenvironment, we collected conditioned medium from LPS‐stimulated macrophages and co‐cultured it with rBMSCs in osteogenic induction medium. RNA sequencing revealed extensive gene expression changes, with 2328 genes significantly upregulated and 2993 downregulated in the HIPPE‐QU‐750 group compared to controls (**Figure** **8**a). The key osteogenic genes, including *Runx2, Bmp2, Spp1, Opn3*, and *Hif‐1α*, were significantly upregulated (Figure 8b), consistent with our qRT‐PCR results (Figure 7e–i). GO enrichment analysis indicated that differentially expressed genes were significantly enriched in biological processes related to extracellular matrix interactions, calcium ion binding, integrin and collagen binding, cell adhesion, and osteoblast differentiation (Figure 8c,d). KEGG pathway and GSEA pathway analysis further highlighted activation of osteogenesis‐associated pathways—particularly MAPK signaling—as well as pathways involved in glucose metabolism and glycolysis (Figure 8e–g). In line with this, glycolytic regulators such as *Glut1*, *Hk2*, and *Pkm* were markedly upregulated (Figure S7b, Supporting Information). GSEA results also showed significant upregulation of TCA cycle and ATP synthesis pathways, underscoring HIPPE‐QU‐750′s role in regulating reprogramming cellular energy metabolism to support osteogenesis (Figure S7c,d, Supporting Information).

Consistent with these transcriptomic findings, Figure 8h demonstrated that HIPPE‐QU‐750 significantly increased the expression of key factors involved in osteogenic signaling factors including *Mapk, Akt, Bmp2, Alp*, *Hif‐1α*, and *Runx2*. This outcome aligns with a mechanism whereby MAPK activation enhances glycolysis‐TCA cycle coupling to supply energy for bone formation and stabilizes HIF‐1α, thereby facilitating osteogenic differentiation.^[^ ^45^ ^]^ Western blot analysis confirmed that HIPPE‐QU‐750 activated MAPK signaling pathways, evidenced by increased expression of GLUT1, ERK, and AKT and stabilization of HIF‐1α. This positive feedback loop enhanced glycolysis and mitochondrial metabolism, thereby creating a nutrient‐rich environment conducive to osteogenesis. It also promoted the expression of critical osteogenic markers, such as RUNX2 (Figure 8i). PPI network analysis further identified HIF1α, GLUT1, and AKT3 as key hubs with extensive interactions bridging metabolism‐related proteins (HK2, PKM2) and osteogenic regulators (RUNX2, BMP2). Moreover, multiple interactions were observed among these osteogenic and metabolic factors (RUNX2, BMP2, AKT3, MAPK12, ATP, HK2, and PKM), highlighting a highly interconnected regulatory network (Figure S7e, Supporting Information). Furthermore, HIPPE‐QU‐750 downregulated immune‐regulatory pathways, including JAK‐STAT, TNF, and p53 signaling (Figure S7a, Supporting Information), which were typically associated with inflammation and osteoclast activity. The suppression of these pathways suggested that HIPPE‐QU‐750 alleviated macrophage‐mediated inflammation, thereby creating a favorable immune microenvironment for osteogenesis.

In conclusion, HQ enhanced glucose metabolism and activated integrin–ECM and MAPK signaling pathways, leading to HIF‐1α stabilization and upregulation of osteogenic proteins. These mechanisms promoted osteogenic differentiation by meeting cellular energy demands and stimulating osteogenic gene expression (Figure 8j).

### 2.9 In Vivo Effect of Bone Regeneration and Periodontal Inflammatory Response

**Figure 9:**
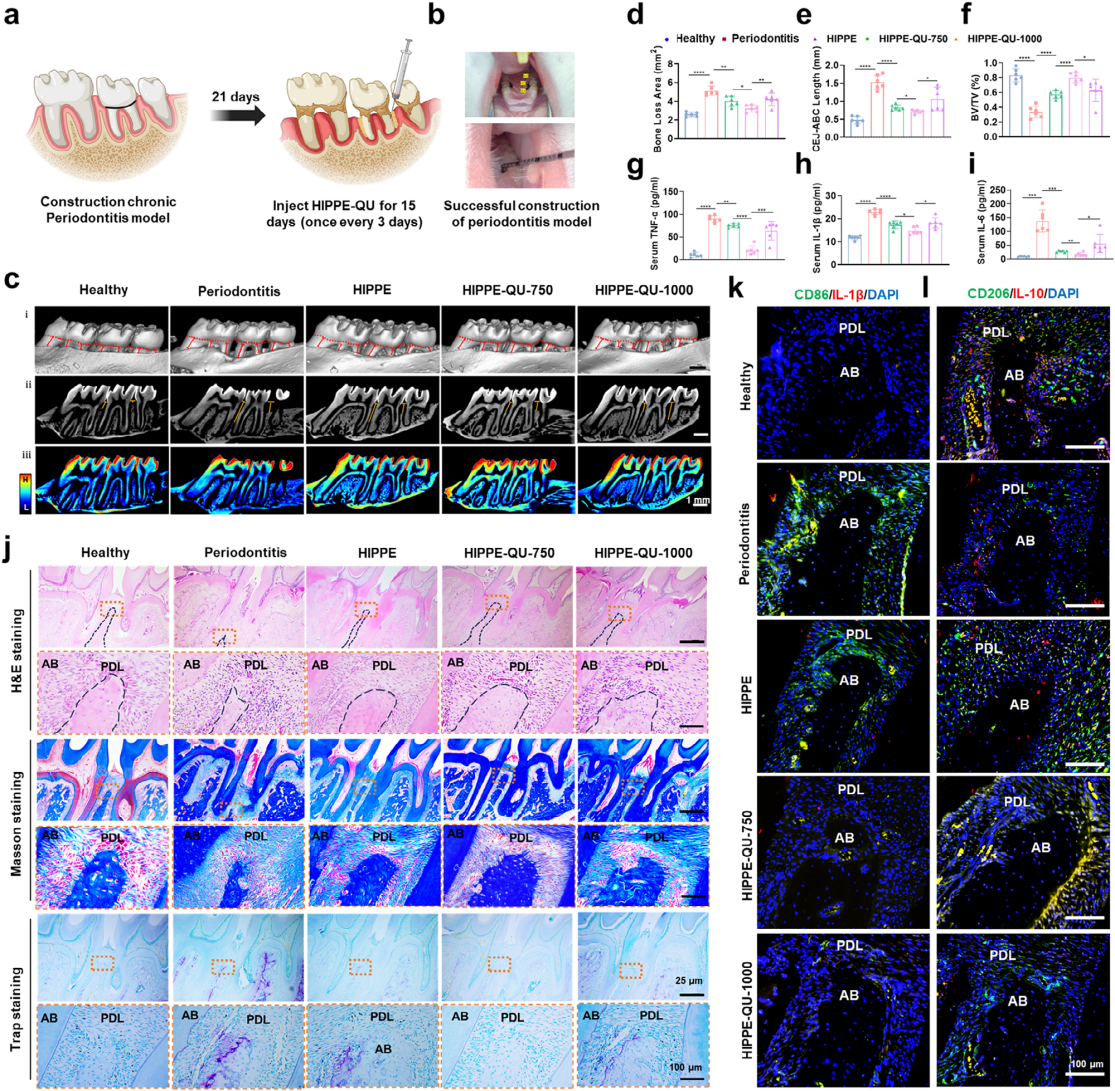
In vivo effect of bone regeneration and periodontal inflammatory response. a,b) Schematic of rat periodontitis model induction and experimental treatment regimen (PBS, HIPPE, HIPPE‐QU‐750, HIPPE‐QU‐1000 injection). c) Micro‐CT analysis of periodontal bone loss (scale bar, 1 mm). (i, ii) Representative cross‐sectional (i) and bucco‐palatal sagittal (ii) views showing the cementoenamel junction (CEJ, dashed red) and alveolar bone crest (ABC, solid red), with the vertical CEJ‐ABC distance indicated by a yellow line in (ii). (iii) 3D reconstruction images displaying bone density variations (high‐density regions in red) and alveolar crest sharpness (heatmap; scale from high (H) to low (L)). d–f) Micro‐CT quantification of bone loss: d) bone loss area; e) CEJ‐ABC length; f) BV/TV. g‐i) ELISA quantification of pro‐inflammatory cytokines in serum: g) TNF‐α; h) IL‐1β; i) IL‐6. j) Histological assessment of AB by H&E, Masson's trichrome, and TRAP staining. The yellow box indicates the AB region between the first and second molars; the alveolar bone crest is outlined by black dashed lines. AB, alveolar bone; PDL, periodontal ligament. k) Immunofluorescence staining of macrophage polarization markers in periodontal tissue: M1 (CD86 [green]; IL‐1β [red]) in k); M2 (CD206 [green]; IL‐10 [red]) in l). Data are expressed as mean ± standard deviation (s.d.) (n = 6). *P < 0.05; **P < 0.01; ***P < 0.001; ****P < 0.0001; ns, not significant.

Encouraged by our in vitro findings that HIPPE‐QU promoted osteogenic differentiation and derived macrophages toward an anti‐inflammatory M2 phenotype; we next evaluated its therapeutic efficacy in vivo using a rat periodontitis model. Periodontitis model was induced by administering *Porphyromonas gingivalis* lipopolysaccharide (P.g‐LPS) and ligating the maxillary second molars for three weeks (**Figure** **9**a,b). Treatments (20 µL of PBS, HIPPE, HIPPE‐QU‐750, or HIPPE‐QU‐1000) were injected into periodontal pockets on days 0, 3, 6, 9, and 12. On day 15, micro‐computed tomography (Micro‐CT) of the maxillae revealed pronounced alveolar bone loss in untreated periodontitis, evidenced by an increased cementoenamel junction–alveolar bone crest (CEJ–ABC) distance compared to healthy controls (Figure 9c). Treatment with HIPPE or HIPPE‐QU markedly preserved alveolar bone, yielding shorter CEJ–ABC distances than the untreated group, with HIPPE‐QU‐750 showing the most pronounced bone protection (Figure 9c). Quantification confirmed that the HIPPE‐QU‐750 group exhibited the greatest reduction in bone loss area and CEJ–ABC distance among the treatments (Figure 9d,e). Moreover, the bone volume fraction (BV/TV) in HIPPE‐QU‐750–treated rats nearly recovered to healthy levels, surpassing the other treatment groups (Figure 9f). Consistent with these bone regenerative effects, enzyme‐linked immunosorbent assays (ELISAs) showed that periodontitis sharply elevated pro‐inflammatory cytokines (TNF‐α, IL‐1β, IL‐6), whereas HIPPE and HIPPE‐QU treatment significantly reduced their levels, with HIPPE‐QU‐750 nearly restoring cytokine concentrations to normal (Figure 9g–i).

Histological analyses corroborated these findings: H&E and Masson staining of diseased periodontal tissue revealed severe epithelial disruption, disorganized collagen fibers, dense inflammatory infiltrates, and extensive bone resorption (Figure 9j). In treated groups, periodontal architecture was largely preserved—periodontal ligament fibers were denser, the epithelium was intact and proliferative, inflammation was markedly diminished, and new bone formation was evident—with HIPPE‐QU‐750 achieving the most substantial tissue recovery. Tartrate‐resistant acid phosphatase (TRAP) staining indicated abundant osteoclast activity in untreated periodontitis, which was greatly attenuated by HIPPE and HIPPE‐QU, with the strongest suppression observed in the HIPPE‐QU‐750 group (Figure 9j). These outcomes were consistent with GO and KEGG pathway analyses showing that HIPPE‐QU‐750 downregulated osteoclast differentiation pathways and promoted anti‐inflammatory macrophage polarization (Figure 6d,e). Immunofluorescence further demonstrated that macrophage polarization in vivo shifted in response to therapy: periodontitis lesions were dominated by M1 macrophages (high CD86 and IL‐1β, low CD206 and IL‐10), whereas HIPPE‐QU treatment skewed macrophages toward the M2 phenotype. HIPPE‐QU‐750 restored M2 macrophage marker expression (CD206, IL‐10) to levels comparable to healthy tissue (Figure 9k), mirroring the polarization trends observed in our cell culture studies (Figure 5a–f; Figure S4a,b, Supporting Information). H&E staining of major organs (heart, liver, spleen, lung, and kidney) showed no treatment‐related histopathological abnormalities or inflammation, confirming the excellent in vivo biocompatibility of both HIPPE and HIPPE‐QU (Figure S8, Supporting Information). Finally, under simulated salivary conditions, a distinct emulsion layer and droplet structures of HIPPE‐QU remained clearly observable at 72 h, indicating that the system maintained overall structural stability without complete demulsification. Only droplet aggregation was noted, suggesting that the emulsion structure remained intact throughout the drug release period. This observation further supports the sustained release behavior described earlier (Figures S9 and S10, Supporting Information). Collectively, these results demonstrated that HIPPE‐QU‐750 was a safe and highly effective therapeutic platform for periodontitis.

## 3 Discussion

During the progression of periodontitis, the regulation of macrophage polarization plays a critical role, characterized by markedly enhanced macrophage glycolysis and ATP metabolism that are partly mediated by the HIF‐1 signaling pathway.^[^ ^26^ ^]^ Targeting this immune imbalance, we developed a multifunctional injectable HIPPE‐QU platform that achieves comprehensive treatment of periodontitis lesions by metabolically reprogramming macrophages and promoting tissue regeneration. This system was prepared via a one‐step homogenization method with an internal phase volume fraction as high as 75%, enabling efficient loading of the hydrophobic drug QU. HIPPE‐QU was co‐stabilized by GNPs and HA‐Cu, forming a gel‐like emulsion with excellent structural stability and injectability. The hydrophobic QU in the oil phase chelated with Cu^2^⁺ and Ca^2^⁺ at the oil–water interface, enabling controlled sustained release and pH‐responsive accelerated release in the acidic inflammatory microenvironment. The nanoparticle‐formed interfacial layer provided an effective steric barrier, stabilizing and protecting QU and overcoming its limitations of poor solubility, low bioavailability, and chemical instability.^[^ ^11^ ^]^ By implementing partitioned loading in the dispersed phase, interfacial layer, and internal phase, HIPPE‐QU simultaneously exhibited synergistic antibacterial and immunomodulatory effects. On the one hand, HIPPE‐QU eradicated the local infection, removing the source of persistent immune stimulation and thereby significantly alleviating periodontal inflammation; on the other hand, the sustained release of QU and Cu^2^⁺ directly reprogrammed macrophage metabolism by modulating key intracellular pathways (such as HIF‐1–mediated glycolysis and controlled ROS generation), which shifted macrophage polarization from a pro‐inflammatory M1 phenotype to an anti‐inflammatory M2 phenotype and reshaped the immune microenvironment to favor tissue regeneration. This two‐pronged mechanism, combining pathogen elimination with direct immune cell modulation, worked in tandem to control infection and inflammation while enhancing the osteogenic activity of rBMSCs, ultimately promoting the repair and regeneration of periodontal tissues.

HA‐Cu possesses both antibacterial and osteogenic properties.^[^ ^19^ ^]^ However, when used alone, HA‐Cu particles exhibit poor interfacial wettability, hindering their migration to the oil–water interface and thereby limiting their ability to stabilize the HIPPE. Nanoparticles of intermediate wettability are considered a key factor in stabilizing HIPPEs.^[^ ^46^ ^]^ The GNPs used here exhibited excellent deformability and surface wettability, effectively stabilizing HIPPEs.^[^ ^23^ ^]^ When GNPs were used in combination with HA‐Cu, both types of particles anchored efficiently at the oil–water interface, enabling controlled release of Cu^2^⁺ for antibacterial effects while simultaneously reducing the cytotoxic risk associated with free Cu^2^⁺. The dual stabilizers (GNPs and HA‐Cu) regulated the emulsion's interfacial wettability and hydrophilic–hydrophobic balance, thereby preserving the emulsion's high internal phase and multi‐phase synergistic structure. As a result, the drug‐loaded emulgel was retained at the lesion site for an extended period, establishing a dual barrier that not only prevented external pathogenic invasion (antibacterial barrier) but also provided a physical foundation for controlled drug release and the regulation of cellular and molecular signals (immune barrier). Thus, the dual‐particle stabilization mechanism enhanced both the structural stability and the biological functionality of the emulsion. Additionally, the flavonoid QU contains multiple phenolic hydroxyl groups,^[^ ^47^ ^]^ which impart a strong oxygen‐coordination ability, enabling chelation with Cu^2^⁺ and Ca^2^⁺ at the interface.^[^ ^48^ ^]^ Such interfacial chelation promoted the migration and anchoring of HA‐Cu nanoparticles at the interface, forming a stable droplet shell structure that greatly improved the emulsion's physical stability and allowed for more controlled, prolonged drug release. When the emulgel encountered the acidic microenvironment of periodontitis lesions, its structure broke down, accelerating the release of QU. Simultaneously, the weakening of QU–Cu^2^⁺/Ca^2^⁺ chelation promoted the release of Cu^2^⁺ and Ca^2^⁺, thereby achieving on‐demand, pH‐responsive delivery.

The pronounced anti‐inflammatory effect of HIPPE‐QU is likely attributable to its potent ROS‐scavenging capability. It is well known that LPS stimulation can induce macrophages to produce excessive ROS, which in turn activates M1 macrophages and promotes the release of pro‐inflammatory factors, thereby exacerbating the inflammatory response.^[^ ^41^, ^49^ ^]^ HIPPE‐QU co‐releases Cu^2^⁺ and QU, both of which possess ROS‐scavenging activity. Together, they significantly lowered ROS levels in the inflammatory microenvironment, attenuated mitochondrial damage, and restored mitochondrial function under oxidative stress. This antioxidant action effectively dampened the amplification of inflammatory signaling and helped suppress excessive M1 polarization. Meanwhile, our results showed that HIPPE‐QU treatment promoted macrophage polarization toward the M2 phenotype and downregulated the expression of various pro‐inflammatory mediators, thereby improving the local immune microenvironment to favor tissue repair. Under hypoxic or LPS‐stimulated conditions, HIF‐1α is a central regulator driving M1 macrophage polarization.^[^ ^50^ ^]^ HIF‐1α upregulates key glycolytic enzymes (e.g., HK1/2, PKM2), thereby promoting glycolysis‐dependent metabolic reprogramming in macrophages.^[^ ^51^ ^]^ This leads to an accumulation of metabolic intermediates such as lactate and succinate, which further activate inflammatory pathways and amplify the pro‐inflammatory effects of M1 macrophages.^[^ ^50^ ^]^ In our study, transcriptomic sequencing and western blot results corroborated this mechanism: HIPPE‐QU significantly suppressed HIF‐1α expression and the activity of its downstream glycolytic pathway, thereby blocking the tendency of macrophages to undergo M1 polarization at the metabolic level.

Apart from its immunomodulatory effects, this system effectively counteracted the inhibitory influence of the inflammatory microenvironment on osteogenesis, and this effect was closely tied to its macrophage‐regulating actions. By guiding macrophages to polarize toward M2 and remodeling cellular metabolism, HIPPE‐QU creates an immuno‐metabolic microenvironment conducive to tissue regeneration.^[^ ^43^, ^52^ ^]^ In this M2 macrophage‐dominated environment, rBMSCs are shielded from the deleterious effects of excessive inflammatory factors, allowing them to maintain and even enhance their osteogenic differentiation potential.^[^ ^53^ ^]^ In HIPPE‐QU‐treated rBMSCs, the MAPK signaling pathway—a key regulator of osteogenesis‐related gene expression—was significantly activated. At the same time, metabolic reprogramming was evident as enhanced glucose uptake and glycolytic activity to meet the high energy and biosynthetic precursor demands of bone formation. This optimization of stem cell metabolism under inflammatory stress ensures sufficient energy and substrates for extracellular matrix synthesis, thereby further supporting bone tissue regeneration.^[^ ^27^, ^54^ ^]^ HIPPE‐QU coordinately modulated immune and metabolic pathways at both transcriptional and protein levels, resulting in a comprehensive improvement of the inflammatory microenvironment and providing strong support for the regeneration process.

HIPPE‐QU‐750 exhibited superior overall efficacy in both osteogenesis and anti‐inflammatory performance than HIPPE‐QU‐1000, which can be attributed to its more coordinated drug release profile and refined modulation of the immune microenvironment. As the principal bioactive compound with both anti‐inflammatory and osteoinductive properties, the release rate of QU directly determines the timeliness and effectiveness of immune regulation and bone formation. Our results showed that HIPPE‐QU‐750 released over 20 µg more QU within 72 h than HIPPE‐QU‐1000, effectively avoiding the delayed release caused by excessive chelation among QU, Cu^2^⁺, and Ca^2^⁺ ions. This more prompt and sufficient release of QU promoted macrophage polarization toward the M2 phenotype, thereby creating an immuno‐metabolic environment conducive to bone regeneration. In this reparative immune milieu, rBMSCs were protected from persistent inflammatory insults and retained or even enhanced their osteogenic differentiation capacity. Meanwhile, the MAPK signaling pathway was activated, and cellular glucose metabolism was enhanced, ensuring adequate energy supply and biosynthetic precursors for matrix production. Therefore, HIPPE‐QU‐750, by fine‐tuning quercetin release kinetics, synergistically regulates immune responses and stem cell metabolism, endowing it with superior regenerative performance.

Interestingly, HIPPE‐QU exhibited cell‐type‐specific regulation of HIF‐1α: it was downregulated in macrophages but upregulated in BMSCs. Although this observation may initially seem paradoxical, it reflects the well‐known context‐dependent nature of signaling pathways. Many canonical pathways, such as NF‐κB, PI3K/Akt, and TGF‐β, can drive distinct—even opposing—biological outcomes in different cellular environments because each cell type possesses unique receptor repertoires, transcriptional co‐factors, and protein interaction networks that shape the ultimate signaling output.^[^ ^55^ ^]^ In macrophages, HIF‐1α is typically stabilized under conditions of chronic inflammation and oxidative stress, thereby maintains a pro‐inflammatory phenotype.^[^ ^56^ ^]^ By mitigating oxidative stress and modulating the immune microenvironment, HIPPE‐QU decreased HIF‐1α stabilization, which contributed to the resolution of inflammation. In contrast, in BMSCs, extensive evidence indicates that HIF‐1α serves as a critical transcription factor promoting osteogenic differentiation by directly regulating osteogenesis‐related genes and metabolic reprogramming required for differentiation.^[^ ^57^ ^]^ Thus, the upregulation of HIF‐1α observed in BMSCs following HIPPE‐QU treatment was consistent with its established pro‐osteogenic role. Taken together, these results illustrated that the same signaling axis could exert divergent effects in different cell types, and advanced biomaterials such as HIPPE‐QU may exploit this regulatory plasticity to achieve multifunctional outcomes. In this case, HIPPE‐QU simultaneously promoted the resolution of inflammation in macrophages while enhancing osteogenesis in BMSCs, which highlighted its potential as a context‐responsive therapeutic platform.

In summary, the injectable HIPPE‐QU platform is engineered with a high‐internal‐phase emulsion architecture, dynamic interfacial synergy, and mechanical self‐healing capability, enabling efficient drug loading and controlled multi‐stage release. The multi‐faceted design allows active components to be co‐loaded in distinct emulsion compartments—HA‐Cu in the dispersed phase, GNPs (with HA‐Cu and chelation complexes) at the interface, and QU in the internal phase—thereby achieving synergistic antimicrobial and immunomodulatory effects that finely tune immune responses, exhibit robust anti‐inflammatory and antibacterial activity, and significantly enhance osteogenesis and periodontal regeneration. Collectively, these findings demonstrate that the HIPPE‐QU platform represents a novel and effective strategy for periodontal tissue regeneration and repair, with significant potential for clinical translation in chronic inflammatory diseases such as periodontitis.

## 4 Experimental Section

### Ethical Statement

This study adheres to all relevant ethical regulations. All animal experiments received approval from the Institutional Animal Care and Use Committee of Southwest Jiaotong University (SWJTU‐2103‐024). Additionally, this study was approved by the Ethics Committee of West China College of Dentistry (reference number WCHSIRB‐D‐2022‐457), and written informed consent was obtained from all participants.

### Preparation of GNPs

The preparation methods for GNPs had been previously detailed in the earlier work.^[^ ^16^ ^]^ In brief, 50 mgmL^−1^ of gelatin solution was first prepared, and then a certain volume of acetone was added to precipitate high‐molecular‐weight gelatin. After that, the sediments were collected and redissolved in distilled water with the pH adjusting to be 12.0 using 2 M NaOH. Again, acetone was added dropwise to trigger the desolvation of gelatin molecules. After stirring for 10 min, glutaraldehyde accounting for 5% of the gelatin content was added to cross‐link the gelatin aggregates, followed by continued stirring at 50 °C for 3 h. Finally, the mixture was centrifuged at 10000 g at 4 °C for 30 min. The collected GNPs were then redispersed in distilled water, and residual acetone was removed by slow vaporization.

### Synthesis of HA‐Cu

HA‐Cu was synthesized using a hydrothermal method.^[^ ^58^ ^]^ A specified amount of CuSO_4_ and Ca (OH)_2_ was dissolved in 200 mL of RO water, ensuring a molar ratio of (Ca + Cu)/P was 1.67. The mixture was thoroughly stirred until a uniform suspension was formed, during which polyethylene glycol was added. Next, 100 ml of H_3_PO_4_ solution (0.3 M) was added dropwise to the suspension, and the reaction was allowed to proceed for 4 h followed by aging for 24 h. The lower suspension was collected and washed twice by centrifugation (Thermo Scientific, Heraeus Megafuge 8R) with anhydrous ethanol and RO water. The resulting mixture was dried, ground, and calcined to obtain HA‐Cu.

### Observation of GNPs and HA‐Cu, Microstructure

The microstructures of GNPs and HA‐Cu were observed using SEM (Gemini 300, Zeiss, Germany). A drop of the diluted GNPs dispersion was applied to a silica wafer and allowed to air‐dry for 12 h. Before observation, all samples were sputtered with gold for 3 min in an argon atmosphere. The morphologies of HA‐Cu were examined under SEM using a similar procedure.

### Preparation of PE‐QU, HIPPE, and HIPPE‐QU

Dissolve QU at concentrations of 750 and 1000 µg mL^−1^ in MCT oil to prepare the oil‐phase solutions. Disperse GNPs and HA‐Cu into the aqueous phase at a 10:5 ratio. Then, add the oil phase containing 750 µgmL^−1^ QU, which accounts for 60% of the total volume, dropwise into the aqueous phase, and emulsify using a high‐shear mixer at an appropriate speed until a stable Pickering emulsion was formed, yielding PE‐QU‐750. Next, add a QU‐free oil phase, accounting for 75% of the total volume, into the aqueous phase under the same emulsification conditions to obtain HIPPE. Finally, add the oil phases containing 750 and 1000 µg mL^−1^ QU, each accounting for 75% of the total volume, into the aqueous phase and emulsify to obtain HIPPE‐QU‐750 and HIPPE‐QU‐1000, respectively.

### Injecting and Molding Tests

PE‐QU and HIPPE‐QU were drawn into a 1 mL syringe and extruded through the needle to simulate writing. The injectability and stability of HIPPE and PE over time were observed and recorded by filming a video.

### Observation of PE‐QU, HIPPE, and HIPPE‐QU Microstructure by CLSM

The microstructures of freshly prepared emulsions were observed using a Leica STELLARIS CLSM (Stellaris 5 WLL, Leica, Germany). The diluted emulsions were placed on a coverslip and were captured by exciting at a wavelength of 543 nm using and bright‐field microscope. The droplet size distribution of the emulsions was determined from the CLSM images using the Mosaic2.4 software.

### Observation of HIPPE Microstructure by SEM

The MCT internal phase of HIPPE was replaced with paraffin, and the structure of HIPPE was scanned by SEM.

### Stability of HIPPE and HIPPE‐QU

The stability of the droplet structure of HIPPE and HIPPE‐QU at pH values of 3, 5, and 7.5 was assessed using an optical microscope.

### Determination of QU‐Ca, QU‐Cu Chelation in HA, HA‐Cu

Solutions of HA and HA‐Cu (2 mgmL^−1^) were prepared in deionized water. Mixtures of HA‐QU and HA‐Cu‐QU (1 mgmL^−1^) were obtained by mixing HA or HA‐Cu with QU in a 1:1 ratio. The mixtures were incubated at 37 °C for 24 h, and their absorption spectrum were measured using a UV–vis spectrometer (Shimadzu UV‐1800, Japan). HA, HA‐Cu, and QU solutions (1 mg mL^−1^) served as controls.

### In Vitro QU Release Study

A total of 1 g of HIPPE‐QU was weighed into a container, followed by the addition of 5 mL of ethyl acetate. The container was incubated at 37 °C and 100 rpm. At 0.5, 1, 1.5, 2, 3, 4, 5, 6, 8, 10, 12, 24, 30, 36, 48, and 72 h, 100 µL of solution was removed and replaced with 100 µL of ethyl acetate. The extracted 100 µL solution was diluted with 600 µL of ethyl acetate and analyzed at 370 nm using a UV‐visible spectrometer (Shimadzu UV‐1800 UV–vis, Japan). The cumulative release of QU was calculated using the standard curve obtained from the absorbance data, and the cumulative percentage release of QU was calculated using the following formula (ER).

(1) $$E_{R}=\left(\frac{V_{e}\sum_{i\;=1}^{n-1}C_{i}+V_{0}C_{n}}{m_{i}}\right)\times 100$$

### In Vitro Cu Release Study

A total of 0.6 g of HIPPE and HIPPE‐QU emulsion was weighed into a container, followed by the addition of 6 mL of PBS. The container was incubated at 37 °C and 100 rpm. At 1, 3, 7, 12, 24, and 48 h, 500 µL of solution was removed and replaced with 500 µL of PBS. The extracted 500 µL solution was collected and subsequently analyzed using ICP‐MS (Agilent 7800, USA).

### Rheological Analysis

The rheological behavior of PE‐QU, HIPPE, and HIPPE‐QU was measured using a rheometer (MARS 40, HAAKE, USA). A 500 µL sample of PE‐QU, HIPPE, and HIPPE‐QU was placed on a temperature‐controlled platform, and a 20 mm diameter plate detector was positioned 1000 µm above the platform. The G′ and G′′ of PE‐QU and HIPPE were measured in the frequency range of 0.1–10 Hz and 0.1–100 Hz using angular frequency sweep tests. The rheological behavior of PE‐QU, HIPPE, and HIPPE‐QU was measured in the frequency range of 0.1–100 Hz. Apparent viscosity was tested with a shear rate range from 0.1 to 100 s^−1^, with the frequency fixed at 1 Hz. Self‐healing properties were determined by switching the strain from low (0.1%, 60 seconds) to high (50%, 60 s) for five cycles, recording the changes in G′ and G′′. Viscosity changes during instantaneous shear rate shifts from 0 to 100 s^−1^ and from 100 to 0 s^−1^ were also recorded. All measurements were conducted at 25 °C.

### In Vitro Antibacterial Experiments

10^4^ CFU mL^−1^ P. gingivalis (ATCC 33 277) and S. aureus (ATCC 25 923) were co‐incubated with 10 µL of HIPPE or HIPPE‐QU in a 24‐well plate. P. gingivalis was maintained under anaerobic conditions for 24 h. Bacterial cells were then collected by centrifugation. The resulting bacterial pellets were fixed in a 2.5% glutaraldehyde solution prepared in PBS. A 50 µL aliquot of each fixed bacterial suspension was applied to sterile silicon wafers designed for electron microscopy and air‐dried in a controlled‐temperature oven until the solvent had completely evaporated. Bacterial morphology was examined, and images were captured using a field emission scanning electron microscope (Gemini SEM 360, Germany). To assess the antibacterial activity of HIPPE and HIPPE‐QU formulations, live/dead staining was conducted on S. aureus and P. gingivalis using the Live/Dead BacLight Bacterial Viability Kit (L7012, Thermo Fisher Scientific, USA). Following the manufacturer's protocol, a staining solution was prepared and added to the washed bacterial suspensions. The stained suspensions were then transferred onto microscope slides for observation under a CLSM. Live bacteria stained green with SYTO 9 (excited at 480 nm), while dead bacteria appeared red due to propidium iodide (PI) staining (excited at 490 nm).

### Isolation and Culture of rBMSCs

rBMSCs were cultured in α‐MEM medium (Gibco, USA) supplemented with 10% FBS (BI, 04‐001‐1ACS, China) and 1% Penicillin‐Streptomycin Solution, which was isolated from the femurs and tibias of healthy, wild‐type male SD rats (3 weeks old). rBMSCs were seeded in culture dishes and incubated in a 5% CO_2_ atmosphere at 37 °C. Once the adherent mesenchymal stem cells reached 90% confluency, they were trypsinized, counted, and replated to establish primary cultures.

### Cell Proliferation and Cell Viability

The survival of rBMSCs and MC3T3‐E1(ATCC, CRL‐2595, USA) was cultured in a 48‐well plate using a complete culture medium that included 5 µL of HIPPE or HIPPE‐QU. After 1 and 3 days of culture, assess cell viability following the instructions provided with the MTT reagent (Beyotime, C0009S, China). The optical density (OD) of the medium was read at 560 nm using a Multi‐function Enzyme Labeler (Revvity Victor NIVO). For live/dead cell staining, the cells incubated with calcein‐AM/PI (Beyotime, C2015S, China), and fluorescence images were captured subsequently.

### Hemolysis Testing

For hemolysis testing, 50 µL of HIPPE‐QU formulations were mixed with 800 µL of 2% (v/v) red blood cell (RBC) suspension prepared in PBS, achieving a final volume of 1 mL per sample. The mixtures were gently vortexed to ensure uniform distribution and incubated at 37 °C for designated time points (1 and 3 h). After incubation, the samples were centrifuged at 3000 rpm for 5 min to pellet the intact RBCs. The absorbance of the supernatant was measured at 540 nm using a microplate reader (or spectrophotometer) to assess hemoglobin release. The hemolysis rate (%) was calculated using the following formula:

(2) $$\;\mathrm{Hemolysis}\mathrm{rate}\left(\%\right)=\frac{\mathrm{Apositive}-\mathrm{Anegative}}{\mathrm{Asample}-\mathrm{Anegative}}\times 100\%$$

where Asample is the absorbance of the sample, Anegative is the absorbance of the PBS control (0% hemolysis), and Apositive is the absorbance of the distilled water control (100% hemolysis).

### Immunofluorescence Staining in Vitro

RAW264.7(ATCC, TIB‐71, USA) and rBMSCs were cultured in confocal 24‐well plates. RAW264.7 cells were co‐cultured with 10 µL of HIPPE or HIPPE‐QU, along with 100 ng mL^−1^ LPS. rBMSCs were cultured macrophages‐conditioned medium, with 10 µl of HIPPE or HIPPE‐QU. For RAW264.7 cells, the primary antibodies used were anti‐CD86 (1:200, ab119857, Proteintech, China), anti‐HIF‐1α(1:100, sc‐13515, Santa Cruz Biotechnology, USA), anti‐CD206 (1:200, Cat No.18704‐1‐AP, Proteintech, China), and anti‐IL‐10 (1:200, 60269‐1‐lg, Proteintech, China).The secondary antibodies were Alexa Fluor 488‐Rabbit (1:300, 34106ES60, YEASEN, China) and Alexa Fluor 594‐Mouse (1:500, A‐11037, Thermo Fisher Scientific, USA). For rBMSCs, the primary antibody used was anti‐vinculin (1:200, ab129002, Abcam, USA). The donkey anti‐rabbit IgG H&L (Alexa Fluor 647 conjugate, 1:500, ab150075, Abcam, USA) was added as the secondary antibody. Then the cells were incubated for 2 h at room temperature in the dark. rBMSCs were counterstained with F‐actin (1:200, 40737ES75, YEASEN, China). Finally, nuclei were stained with DAPI (ab1041139, Abcam, USA). CLSM was used to acquire the images, and analysis was performed using Mosaic2.4 software.

### Cellular ROS Scavenging Activity

ROS levels of RAW 264.7 cells were measured using the Reactive Oxygen Species Assay Kit (S0033S) following the kit protocol. The detection was via fluorescence imaging and flow cytometry (Cytoflex, Beckman, USA).

### Detection of MMP

RAW 264.7 cells were stained with JC‐1 to evaluate MMP following the manufacturer's instructions (Beyotime, S0026, China). The change in fluorescence from green (≈514 nm) to red (≈590 nm) was observed using confocal microscopy, indicating mitochondrial depolarization in the macrophages.

### ATP Measurement

Protein concentration of RAW 264.7 cells was measured prior to the ATP assay. Intracellular ATP levels were quantified using the Enhanced ATP Assay Kit (Abscience), following the manufacturer's instructions (Beyotime, S0026, China). ATP concentration was determined by comparing the measured values to a standard ATP curve. Relative light unit (RLU) values were obtained using a luminometer (Revvity Victor NIVO). The ATP concentration of each sample was calculated based on the standard curve, and the intracellular ATP content was normalized to the protein concentration to account for the total protein amount in each sample.

### Osteogenic Differentiation of rBMSCs in Vitro Under Macrophages‐Conditioned Medium

For osteogenic differentiation experiments, rBMSCs cultured with a 1:2 mixture of macrophages‐conditioned medium and osteogenic induction medium. RAW 264.7 cells were stimulated with 100 ng mL^−1^ LPS for 24 h to collect the supernatant as macrophages‐conditioned medium. The osteogenic induction medium was prepared by supplementing the culture medium with 5 mM β‐glycerophosphate, 50 µg mL^−1^ ascorbic acid, and 100 nM dexamethasone. The ability of HIPPE and HIPPE‐QU to promote rBMSCs differentiation was assessed by immunofluorescence staining, ALP staining, ARS staining, and analysis of osteogenesis‐related gene and RNA sequencing.

### ALP and ARS Staining

For ALP staining, rBMSCs were stained with the BCIP/NBT ALP Color Development Kit (Biosharp, BL862B) on day 7. Quantitative ALP analysis was evaluated with an ALP Assay Kit (Cell Biolabs, CA) and normalized to the total protein content determined by a BCA Protein Assay Kit according to the manufacturer's protocol. For ARS staining, rBMSCs were stained with 2% Alizarin Red S solution (ALIR‐10001, China) on day 14. Mineral deposits were quantified by dissolving with 10% cetylpyridinium chloride and measuring optical densities (OD) at 562 nm using a Multi‐function Enzyme Labeler (Revvity Victor NIVO).

### qRT‐PCR

Total RNAs from RAW264.7, rBMSCs, and HGFs were extracted with TRIzol Reagent (Thermo Fisher, USA). Following extraction, RNA was converted to cDNA using the PrimeScript RT Reagent Kit (Takara, Japan). qRT‐PCR was performed on a LightCycler 96 system (Roche, Switzerland, Germany) using SYBR Premix EX Taq (Takara, Japan) to quantify the expression levels of inflammation‐related gene (*Il‐1β*, *Il‐6*, and *Tnf‐α*), osteogenic genes (*Alp*, *Ocn*, *Ocn*, *Runx‐2*, and *Col1a1*), and adhesion and fibrosis‐related genes (*FN1, FAK, ITGB1, COL1A1, and VCL*). Target gene expression levels were normalized to *Gapdh* for consistent and accurate quantification. The sequences of primers are shown in Table S1 (Supporting Information).

### Western Blot

The total protein from RAW264.7 and rBMSCs were harvested by a total protein extraction kit (SAB, PE001‐2, China). western blot was performed per standard protocol. Antibodies used in western blot as follows: mouse anti‐MYD88 antibody (1:2000, 67969‐1‐Ig, Proteintech, China), mouse anti‐mTOR antibody (1:25 000, 66888‐1‐Ig, Proteintech, China), rabbit anti‐AMPK antibody, rabbit anti‐HIF‐1α(1:1000, ab207442, Abcam, USA), mouse anti‐NF‐kB (1:1000, #Q04206, Cell Signaling Technology, USA), mouse anti‐TNF‐α (Proteintech, 60291‐1‐Ig, 1:1000), rabbit anti‐iNOS (1:200, 22226‐1‐AP, Proteintech, China), mouse anti‐NLRP3 (1:2000, 68102‐1‐lg, Proteintech, China), rabbit anti‐IL‐1β (1:1000, ab254360, Abcam), mouse anti‐Tubulin (1:100, sc‐5286, Santa Cruz Biotechnology, USA), IRF4 (1:1000, ab315394, Abcam, USA), mouse anti‐STAT3 (1:1000, #9139, Cell Signaling Technology, USA), rabbit anti‐GLUT1(1:1000, 21829‐1‐AP, Proteintech, China), rabbit anti‐ERK1/2 (1:500, WL01864, Wanleibio), rabbit anti‐p‐ERK1/2 (1:1000, 28733‐1‐AP, Proteintech, China), mouse anti‐AKT(1:100, sc‐5298, Santa Cruz Biotechnology, USA), rabbit anti‐RUNX2(1:200, 20700‐1‐AP, Proteintech, China), and then incubated with HRP (horseradish peroxidase) conjugated secondary antibodies, anti‐rabbit (1:5000, Cat No. SA00001‐2, Proteintech, China) and anti‐mouse (1:5000, Cat No. SA00001‐1, Proteintech, China). The bound antibody was detected using a standard chemiluminescence method with the ChemiDoc Touch Imaging System. (Bio‐Rad, Universal Hood ll, USA).

### Animals and Treatments

Wild‐type male SD rats were sourced from Chengdu Dashuo Animal Co., Ltd., with all rats aged 8 to 12 weeks. They were housed in specific‐pathogen‐free (SPF) conditions, maintained on a 12‐h light/dark cycle, and at a temperature of 22 to 24 °C. The rats were acclimatized to the animal facility for one week prior to being randomly assigned to experimental groups (Healthy, Periodontitis, HIPPE‐QU‐750, HIPPE‐QU‐1000). First, the rats were anesthetized by intraperitoneal injection of pentobarbital sodium (50 mg kg^−1^, Sigma). Subsequently, a 4‐0 silk ligature was placed around the second molar for 3 weeks to induce periodontitis. Once the model was successfully established, a total of 20 µL of HIPPE or HIPPE‐QU was injected into the periodontal area of the second molar over two consecutive weeks for treatment. After completion of the treatment, the rats were euthanized by an overdose of pentobarbital sodium, and the relevant tissue samples were collected for further analysis.

### Micro‐CT Analysis

Rat maxillae were fixed in 4% polymerized formaldehyde for 48–72 h, then scanned and analyzed using a Venus Micro CT (VNC‐102, PingSeng Healthcare, China) at an operating voltage of 50 kV, current of 200 µA, exposure time of 300 ms, and a resolution of 6.0 µm per pixel. 3D reconstruction images of the maxillary samples were acquired to assess the bone volume to tissue volume ratio (BV/TV). Thermal mapping of the crowns and other regions was performed using SCANCO Visualizer software (version 1.1.18.0), with enamel appearing red due to its high density. Bone resorption between neighboring teeth was evaluated by measuring the vertical distance from the CEJ to the ABC using X‐rays. The area between the first and second molars was selected for bone loss analysis.

### ELISA Assay

Blood was collected from the hearts of rats and allowed to clot on ice for 1 h, followed by centrifugation at 3000 rpm for 15 min to obtain serum. The levels of inflammatory factors IL‐6, TNF‐α, and IL‐1β in serum were measured by ELISA kits (RUIXIN BIOTECH, RX302856R/RX302858R/RX302869R).

### Histological Analysis

Heart, liver, spleen, lung, kidney, and maxilla were fixed in 4% polymerized formaldehyde for 48–72 h, embedded in paraffin, and sectioned into 5 µm slices. The sections underwent H&E staining, while the maxilla was also stained using Masson's trichrome and TRAP staining according to standard protocols.

### Immunofluorescence Staining in Vivo

Maxilla were subjected to immunofluorescence staining. The primary antibodies used were anti‐CD86, anti‐IL‐1β (1:50, ab254360, Abcam, USA), anti‐CD206, and anti‐IL‐10. The secondary antibodies were Alexa Fluor 488‐Rabbit and Alexa Fluor 594‐Mouse. Finally, nuclei were stained with DAPI. CLSM was used to acquire the images.

### The Morphological and Structural Changes of the Emulsion in the Salivary Environment

Emulsions were incubated in simulated salivary fluid at 37 °C. Samples were taken at set time points for CLSM imaging and droplet size analysis using Mosaic2.4 to assess emulsion stability and retention under periodontal‐like conditions.

### RNA‐Sequencing

Total RNA from pro‐inflammatory RAW264.7 and BMSCs with or without HIPPE‐QU‐750 was extracted. The RNA samples were sent to OE Biotech, Inc. for sequencing to investigate transcriptomic profiles under different conditions. High‐quality RNA underwent sequencing on the Illumina NovaSeq 6000 platform, and the data were analyzed using DESeq2. Transcripts with a p value < 0.05 and a fold change > 2 were designated as significantly differentially expressed genes. GO analyses was implemented using Metascape (metascape.org) to identify differentially expressed transcripts. KEGG, GSEA, and PPI network interactions were analyzed using the OE Biotech online platform. (https://cloud.oebiotech.com).

### Statistical Analysis

Statistical analyses were performed using SPSS v.22. software (IBM, Armonk, NY, USA). All results were presented as mean ± standard deviation and analyzed by a two‐tailed Student‐test. p < 0.05 was considered statistical significance.

## Conflict of Interest

The authors declare no conflict of interest.

## Author Contributions

H. Z., X. H., and R. C. designed and performed the experiments, analyzed the data, and wrote the manuscript. L.W. collected data. K. ‐L.L., X.P., X.‐Y.C. performed the experiments. T. ‐L.G., J.W, Q.Y., and J. ‐S. L. provided technical assistance. all authors contributed to the interpretation of experiments. H.T. and M.Y.W. provided funding and supervision.

## Supporting information



Supporting Information

Supporting Information

## Data Availability

The data that support the findings of this study are available in the supplementary material of this article.
