# Supplementary material for: Interfacial Coordination‐Engineered Emulgel Modulates the Osteo‐Immune Microenvironment in Periodontitis Treatment Via HIF1 Signaling Pathway‐Induced Metabolic Reprogramming
Source: Adv Sci (Weinh). 2025 Sep 29;12(46):e09998. doi: 10.1002/advs.202509998 (PMC12697898; doi:10.1002/advs.202509998)
Supplement: Supplementary file 1 — Supporting Information [file ADVS-12-e09998-s002.docx]

Supporting Information

**Interfacial Coordination-Engineered Emulgel Modulates the Osteo-Immune Microenvironment in Periodontitis Treatment via HIF1 Signaling Pathway-Induced Metabolic Reprogramming**

*Hui Zhang^1,2,3^, Xiao Huang^1^, Rui Chen^1,4^, Weimin Li^5^, Linke Li^1,2^, Xi Peng^1^, Xinxi Yang^1,2^, Xingyu Chen^1,3^, Tailin Guo^1,3^, Jie Weng^1,3^, Jianshu Li^6^, Quan Yuan^5^, Huan Tan^1,3^*, Mengyuan Wang^1,2,5^**

Author Affiliations:

1 College of Medicine, Southwest Jiaotong University, Chengdu 610031, Sichuan, China.

2 Center of Obesity and Metabolic Diseases, Department of General Surgery, The Third People's

Hospital of Chengdu, Chengdu 610014, P. R. China.

3 Key Laboratory of Advanced Technologies of Materials Ministry of Education, School of Materials Science and Engineering, Southwest Jiaotong University, Chengdu 610031, P. R. China.State

4 Department of stomatology, The Third People's Hospital of Chengdu, The Affiliated Hospital of Southwest Jiaotong University, Chengdu, Sichuan, China.

5 Key Laboratory of Oral Diseases and National Center for Stomatology, National Clinical Research Center for Oral Diseases, West China Hospital of Stomatology, Chengdu 610041, P. R. China.

6 College of Polymer Science and Engineering. State Key Laboratory of Polymer Materials Engineering, Sichuan University, Chengdu 610041, P. R. China.

Correspondence author:

Mengyuan Wang ([wangmengyuan@swjtu.edu.cn](mailto:wangmengyuan@swjtu.edu.cn)), Huan Tan([tanhuan@swjtu.edu.cn](mailto:tanhuan@swjtu.edu.cn))

**Content:**

**Supplementary Fig. 1 Macrophage polarization and metabolism associated with periodontitis.**

**Supplementary Fig. 2 Long-term stability of HIPPE and HIPPE-QU.**

**Supplementary Fig. 3 The average droplet sizes of 60%-PE-QU-750, HIPPE, HIPPE-QU-750 and HIPPE-QU-1000.**

**Supplementary Fig. 4 RNA sequencing analysis of RAW264.7 macrophages co-cultured with HQ under LPS stimulation.**

**Supplementary Fig. 5 HIPPE-QU-750 enhances cell adhesion and repair potential of HGFs under inflammatory conditions.**

**Supplementary Fig. 6 Immunofluorescence images of rBMSCs morphology on HIPPE and HIPPE-QU-750 surfaces.**

**Supplementary Fig. 7 RNA sequencing analysis of rBMSCs co-cultured with HQ in an inflammatory microenvironment.**

**Supplementary Fig. 8 Biocompatibility assessment of HIPPE and HIPPE-QU. H&E staining of heart, liver, spleen, lung, and kidney tissues from treated rats.**

**Supplementary Fig. 9 Morphological of HIPPE-QU formulations in simulated salivary fluid (SSF, pH 6.8). Representative macroscopic and optical microscopy images at 1, 6, 24, and 72 h show phase separation and changes in droplet morphology over time.**

**Supplementary Fig. 10 The droplet size distribution of HIPPE, HIPPE-QU-750, and HIPPE-QU-1000 formulations at 1, 6, 24, and 72 h in simulated salivary fluid (SSF, pH 6.8).**

**Supplementary Table 1** **PCR primer sequences.**

**

**

**Supplementary Fig. 1 Macrophage polarization and metabolism associated with periodontitis.** (a–e) Proportions of M1 macrophage markers HIF-1α (a), NLRP3 (b), and IL-1β (c) were significantly elevated in periodontitis patients compared to healthy controls, with respective values of 46.3%, 31.27%, and 71.64% in patients versus 27.1%, 20.31%, and 50.82% in controls. (d, e) Proportions of M2 macrophage markers IL-10RA (d) and IRF4 (e) were reduced in periodontitis patients, at 27.24% and 6.85%, respectively, compared to 34.12% and 8.84% in controls.


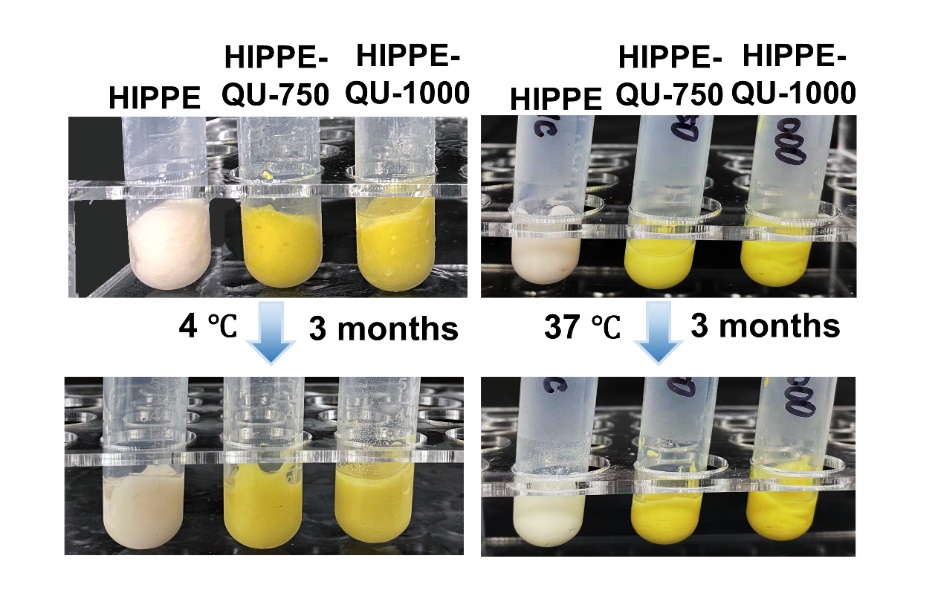


**Supplementary Fig. 2 Long-term stability of HIPPE and HIPPE-QU.** HIPPE and HIPPE-QU maintained structural integrity and phase stability over 3 months of storage at 4 °C and 37 °C.


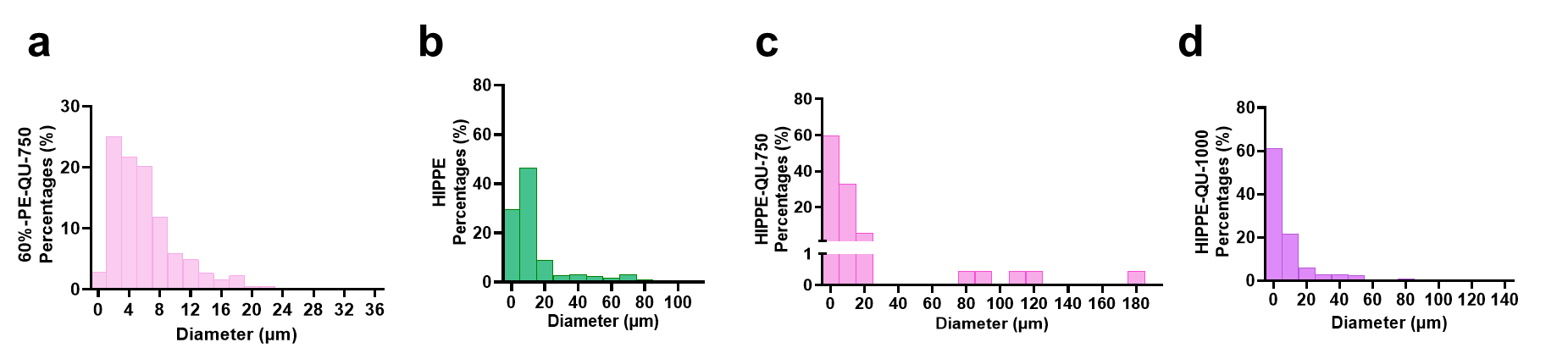


**Supplementary Fig. 3** (a-d) The average droplet sizes of 60%-PE-QU-750 (a), HIPPE (b), HIPPE-QU-750 (c) and HIPPE-QU-1000 (d).


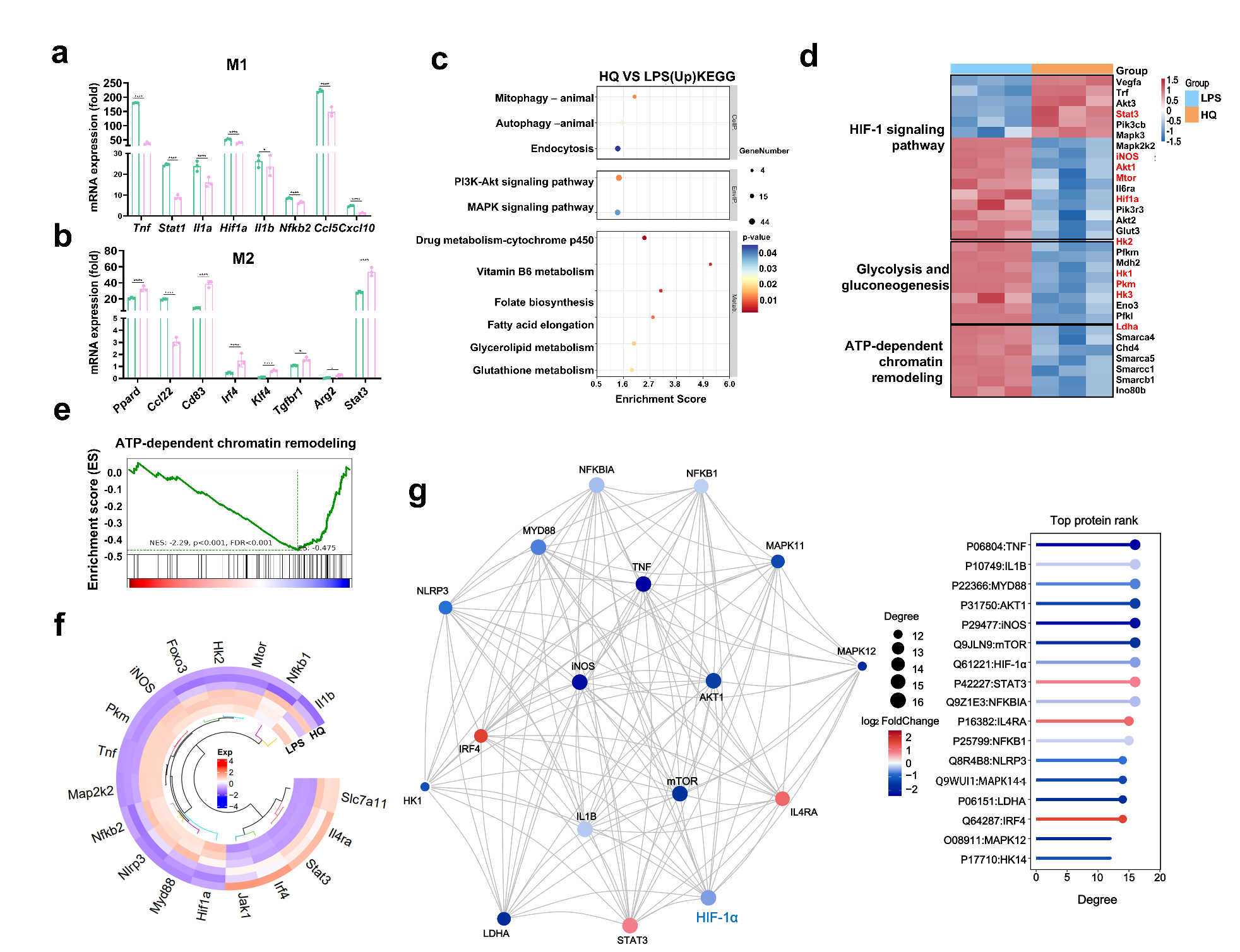


**Supplementary Fig. 4 RNA sequencing analysis of RAW264.7 macrophages co-cultured with HQ under LPS stimulation.** (a, b) HQ treatment markedly downregulated mRNA levels of M1 macrophage markers (a) while upregulating M2 macrophage markers (b). (c) KEGG pathway enrichment analysis revealed the upregulation of key signaling pathways and regulatory mechanisms in the HQ group. (d) Heatmap showing differentially expressed genes in critical pathways, including the HIF-1 signaling pathway, glycolysis and gluconeogenesis, and ATP-dependent chromatin remodeling. (e) GSEA analysis identified ATP-dependent chromatin remodeling as a key downregulated regulatory mechanism following HQ treatment. (f) Circular heatmap illustrating significantly downregulated metabolic and immune-regulatory factors in M1 macrophages. (g) PPI network of differentially expressed genes constructed using the STRING database. Red indicates upregulated genes, and blue indicates downregulated genes, with NF-κB subunits, MYD88, mTOR, and HIF-1α identified as central regulators.


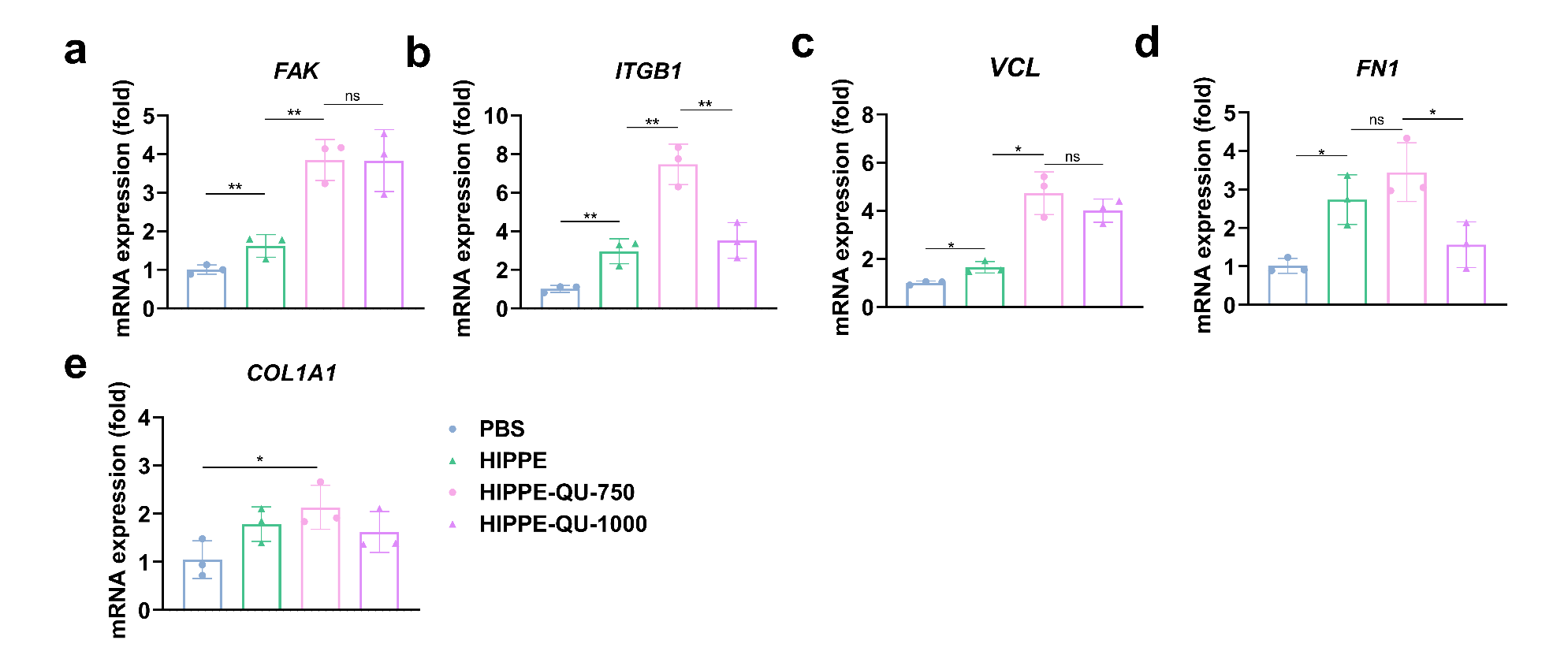


**Supplementary Fig. 5 HIPPE-QU-750 enhances cell adhesion and repair potential of HGFs under inflammatory conditions.** (a-e) HGFs were cultured for 1 day in macrophage-conditioned medium derived from different HIPPE-QU groups. The mRNA expression levels of *FAK* (a), *ITGB1*(b), *VCL*(c), *FN1*(d) and *COL1A1*(e) were assessed using qRT-PCR. Data are presented as mean ± standard deviation (s.d.) (n = 3); **p* < 0.05; ***p* < 0.01; ns, not significant


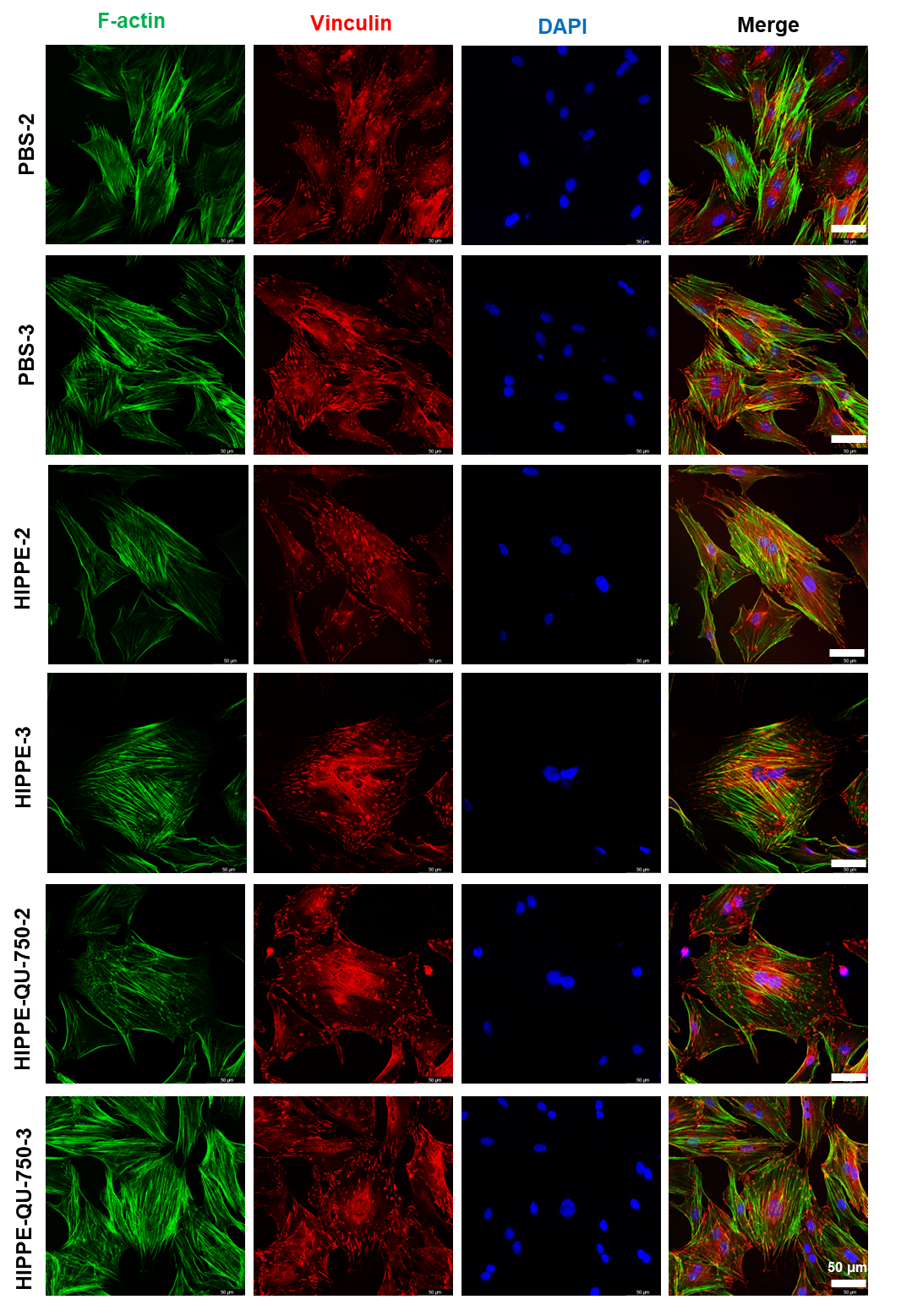


**Supplementary Fig. 6 Immunofluorescence images of rBMSCs morphology on HIPPE and HIPPE-QU-750 surfaces.**

**
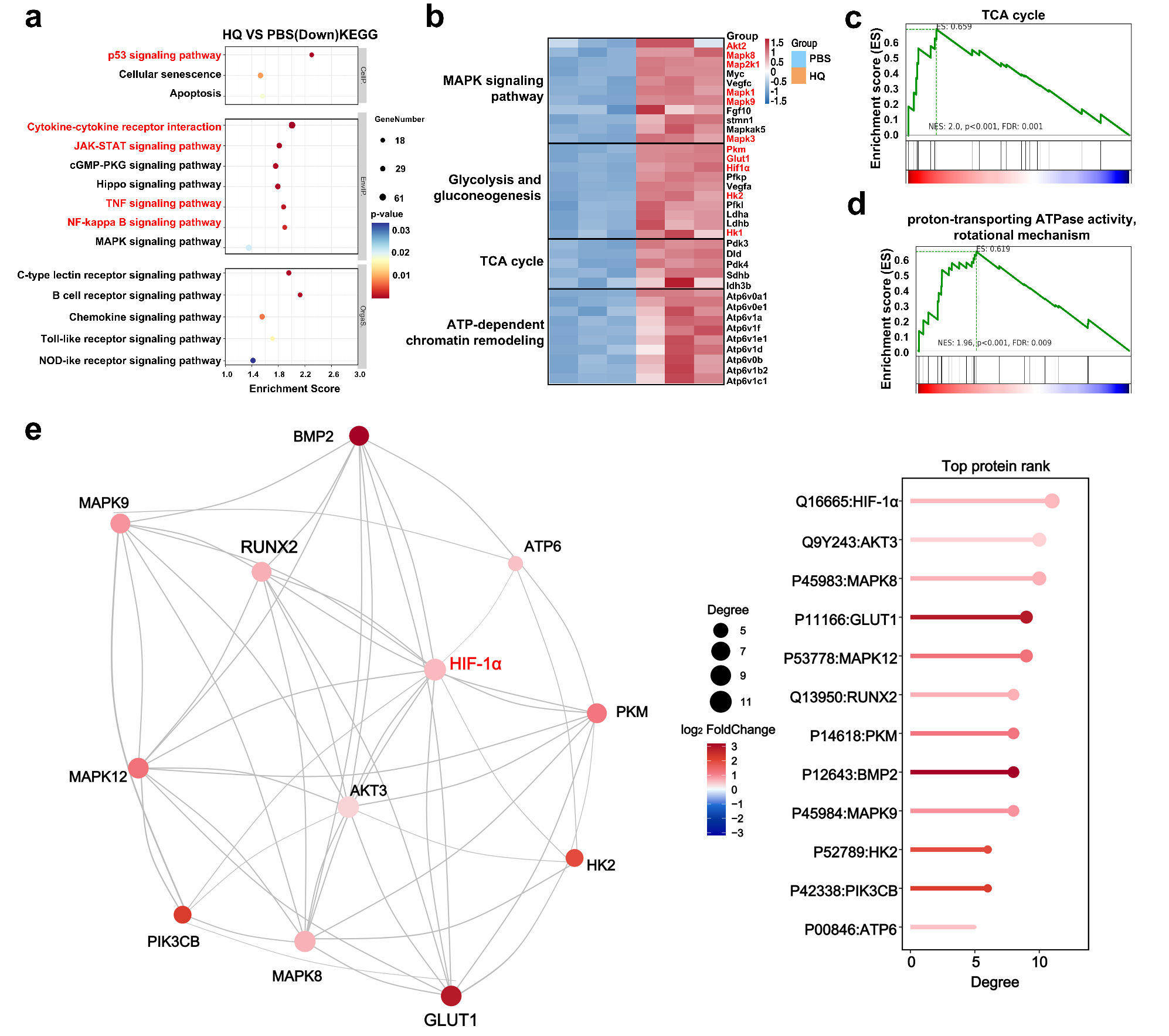
**

**Supplementary Fig. 7 RNA sequencing analysis of rBMSCs co-cultured with HQ in an inflammatory microenvironment.** (a) KEGG pathway enrichment analysis identified significantly downregulated signaling pathways and regulatory mechanisms. (b) Heatmap displaying key upregulated genes involved in critical pathways, including the MAPK signaling pathway, glycolysis and gluconeogenesis, the TCA cycle, and ATP-dependent chromatin remodeling. (c, d) GSEA analysis revealed key upregulated regulatory mechanisms after HQ treatment, including the TCA cycle (c) and proton-transporting ATPase activity (d). (e) PPI network identifying HIF-1α, GLUT1, and AKT3 as key hubs interacting with glycolysis-related proteins (HK2, PKM2) and osteogenesis markers (RUNX2, BMP2). Red nodes represent upregulated genes.

**
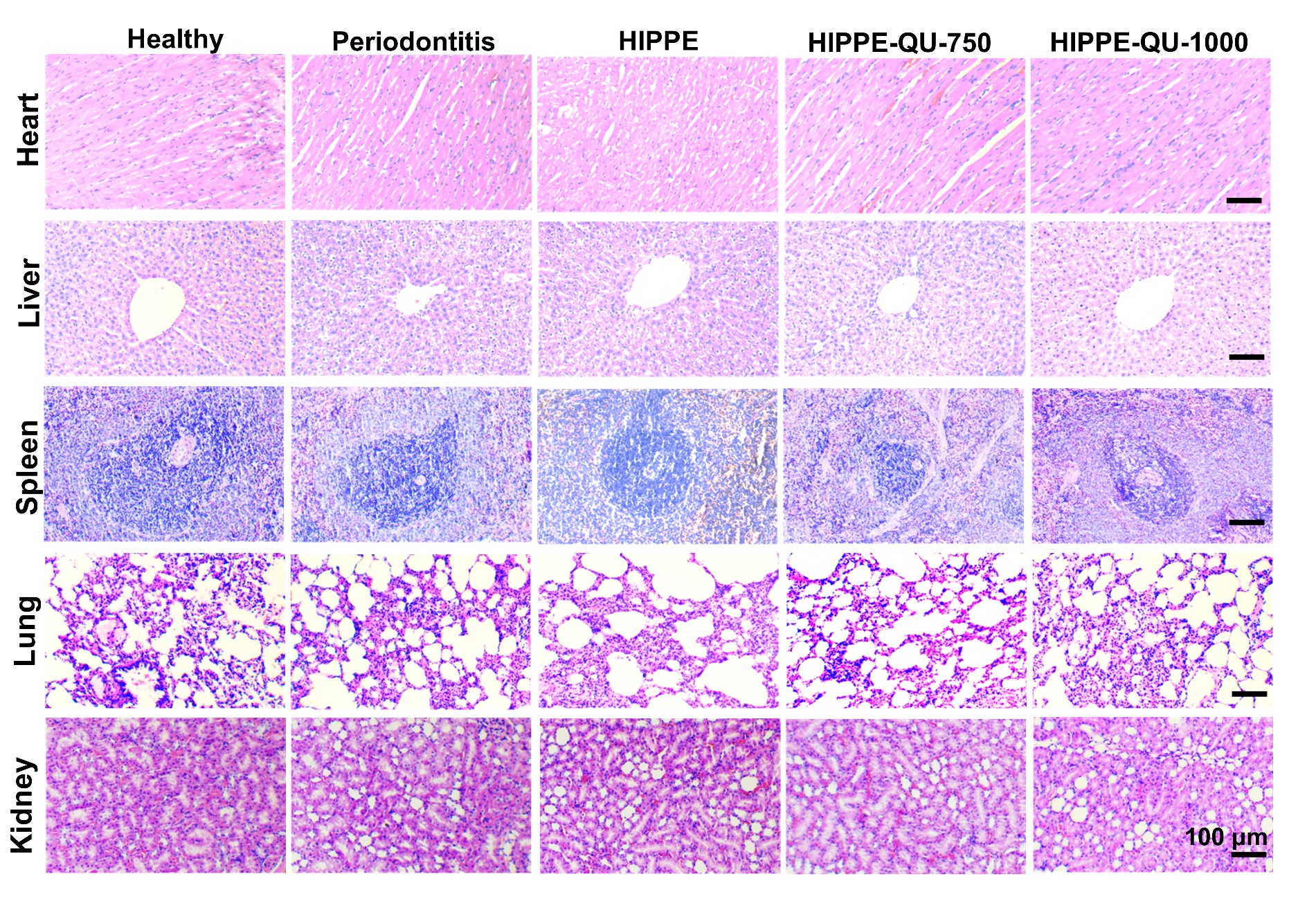
**

**Supplementary Fig. 8 Biocompatibility assessment of HIPPE and HIPPE-QU. H&E staining of heart, liver, spleen, lung, and kidney tissues from treated rats.**

**
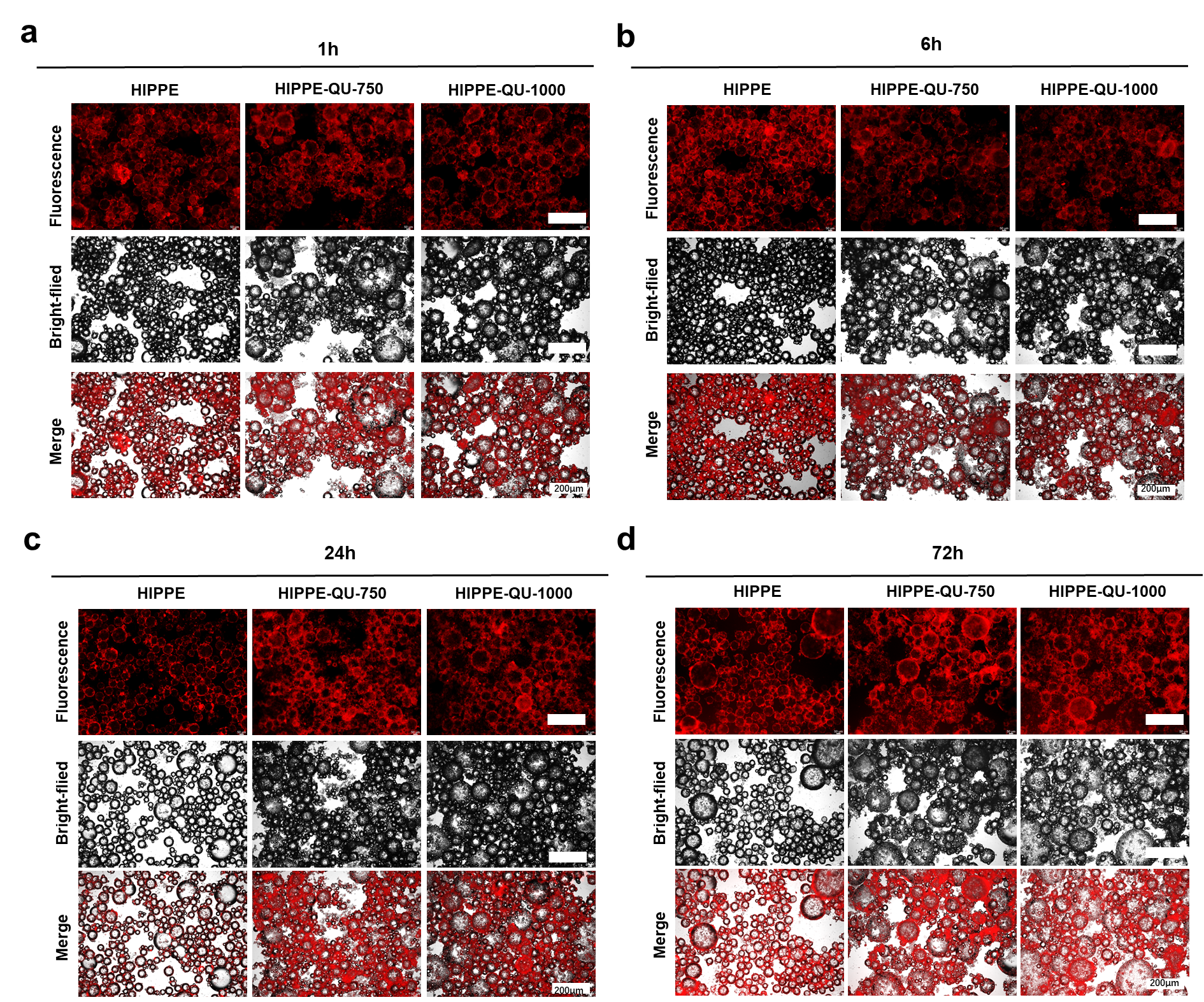
**

**Supplementary Fig. 9. Morphological of HIPPE-QU formulations in simulated salivary fluid (SSF, pH 6.8). Representative macroscopic and optical microscopy images at 1, 6, 24, and 72 h show phase separation and changes in droplet morphology over time.**

**
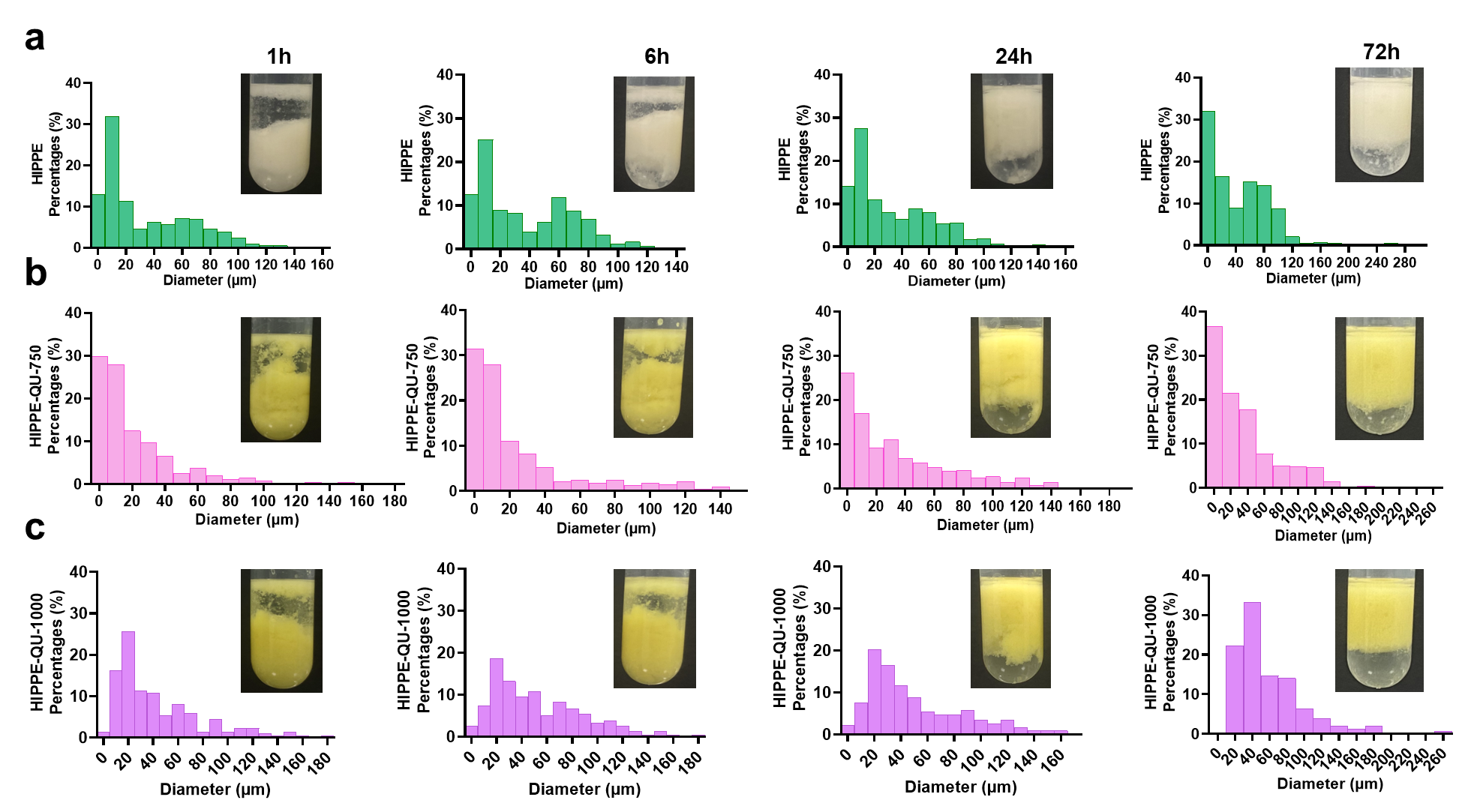
**

**Supplementary Fig. 10. The droplet size distribution of HIPPE, HIPPE-QU-750, and HIPPE-QU-1000 formulations at 1, 6, 24, and 72 h in simulated salivary fluid (SSF, pH 6.8).**

**Supplementary Table 1** **PCR primer sequences.**

| Primers | Forward | Reverse |
| --- | --- | --- |
| *hGAPDH* | TGTGTCCGTCGTGGATCTGA | TTGCTGTTGAAGTCGCAGGAG |
| *hVCL* | CGAATCCCAACCATAAGCAC | CGCACAGTCTCCTTCACAGA |
| *hCOL1A1* | AAGACATCCCACCAATCACC | CGTCATCGCACAACACCTT |
| *hFAK* | CTCCTACTGCCAACCTGGAC | GCCGACTTCCTTCACCATAG |
| *hVCL* | CGAATCCCAACCATAAGCAC | CGCACAGTCTCCTTCACAGA |
| *hFN1* | GACCGAAATCACAGCCAGTAG | CATCTCCCTCCTCACTCAGC |
| *hITGB1* | TGGAGGAAATGGTGTTTGC | CGTTGCTGGCTTCACAAGTA |
| *rGapdh* | TCAACAGCAACTCCCACTCTTCCA | ACCCTGTTGCTGTAGCCGTATTCA |
| *rCol1a1* | CATAAAGGGTCATCGTGGCT | TTGAGTCCGTCTTTGCCAG |
| *rAlp* | CCAACTCTTTTGTGCCAGAGA | GGCTACATTGGTGTTGAGCTTTT |
| *rRunx2* | AACGATCTGAGATTTGTGGGC | CCTGCGTGGGATTTCTTGGTT |
| *rOcn* | GGCGTCCTGGAAGCCAATGTG | GACCAGGAGGACCAGGAAGTCCACGT |
| *rOpn* | GATGGCCGAGGTGATAGTGT | GTGGGTTTCAGCACTCTGGTG |
| *mGapdh* | TGCACCACCAACTGCTTAG | GATGCAGGGATGATGTTC |
| *mIl-6* | CTGCAAGAGACTTCCATCCAGTT | GAAGTAGGGAAGGCCGTGG |
| *mTnf-α* | GAACTGGCAGAAGAGGCACT | AGGGTCTGGGCCATAGAACT |
| *mIl-1β* | GCAACTGTTCCTGAACTCAACT | ATCTTTTGGGGTCCGTCAACT |
